# Strength of *Neisseria meningitidis* binding to endothelial cells requires highly-ordered CD147/β_2_-adrenoceptor clusters assembled by alpha-actinin-4

**DOI:** 10.1038/ncomms15764

**Published:** 2017-06-01

**Authors:** Nawal Maïssa, Valentina Covarelli, Sébastien Janel, Beatrice Durel, Nandi Simpson, Sandra C. Bernard, Liliana Pardo-Lopez, Haniaa Bouzinba-Ségard, Camille Faure, Mark G.H. Scott, Mathieu Coureuil, Philippe C. Morand, Frank Lafont, Xavier Nassif, Stefano Marullo, Sandrine Bourdoulous

**Affiliations:** 1 Inserm, U1016, Department of Infection, Immunity and Inflammation, Institut Cochin, 75014 Paris, France; 2 CNRS, UMR 8104, 75014 Paris, France; 3 Université Paris Descartes, Sorbonne Paris Cité, 75006 Paris, France; 4 Cellular Microbiology and Physics of infection, Center for Infection and Immunity of Lille, Institut Pasteur de Lille, 59000 Lille, France; 5 CNRS, UMR 8204, 59000 Lille, France; 6 Inserm, U1019, 59000 Lille, France; 7 Université de Lille, 59000 Lille, France; 8 Inserm, unité U1151, Institut-Necker-Enfants-Malades, 75015 Paris, France; 9 CNRS, UMR 8253, 75015 Paris, France; 10 Université Paris Descartes, Sorbonne Paris Cité, Faculté de Médecine, 75006 Paris, France; 11 Assistance Publique—Hôpitaux de Paris, Hôpital Necker Enfants Malades, 75015 Paris, France

## Abstract

*Neisseria meningitidis* (meningococcus) is an invasive bacterial pathogen that colonizes human vessels, causing thrombotic lesions and meningitis. Establishment of tight interactions with endothelial cells is crucial for meningococci to resist haemodynamic forces. Two endothelial receptors, CD147 and the β2-adrenergic receptor (β_2_AR), are sequentially engaged by meningococci to adhere and promote signalling events leading to vascular colonization, but their spatiotemporal coordination is unknown. Here we report that CD147 and β_2_AR form constitutive hetero-oligomeric complexes. The scaffolding protein α-actinin-4 directly binds to the cytosolic tail of CD147 and governs the assembly of CD147–β_2_AR complexes in highly ordered clusters at bacterial adhesion sites. This multimolecular assembly process increases the binding strength of meningococci to endothelial cells under shear stress, and creates molecular platforms for the elongation of membrane protrusions surrounding adherent bacteria. Thus, the specific organization of cellular receptors has major impacts on host–pathogen interaction.

## Abstract (web summary)

*Neisseria meningitidis* bacteria bind to host proteins CD147 and β2-adrenergic receptor on the surface of endothelial cells. Here, Maïssa *et al*. show that the two proteins interact with each other forming clusters that increase the binding strength of the bacteria to endothelial cells.

The endothelium of blood and lymphatic vessels is a physiological barrier separating tissues from body fluids and a major target for many pathogenic bacteria^1^. Blood-borne pathogens establish intimate interactions with endothelial cells, triggering local inflammatory responses and coagulation. Pathogens also modify endothelial plasma membrane and intercellular junctions to establish firm adhesion, cross and/or disrupt the endothelial barrier and ultimately invade tissues^1^. Tight interaction with the endothelial cell surface is essential for pathogens to resist the mechanical forces exerted by the blood flow^2^.

Among the pathogens interacting with human vessels, *Neisseria meningitidis* (meningococcus) is potentially one of the most harmful. This obligate human Gram-negative bacterium normally resides in the nasopharynx without affecting the host. However, when meningococci reach the bloodstream they can cause a rapidly progressing fatal septic shock known as *purpura fulminans* and infect the meninges^3^. Meningococcal dissemination through the blood stream relies on the capacity of these bacteria to interact with microvessels and proliferate on the endothelial cell surface to form bacterial aggregates^4^. This vascular colonization precedes dissemination into tissues, including meninges, and promotes deregulated inflammation and coagulation, leading to extensive necrotic purpura in the most severe cases.

Tight interaction of virulent capsulated *N. meningitidis* with vascular cells relies on type IV pili^5^. These long filamentous structures expressed by all clinical isolates^6^ result from the assembly of pilin subunits into helical fibres^7^. The major pilin subunit, PilE, constitutes the fibre scaffold^8^, whereas other less abundant pilins, such as ComP, PilX or PilV, which structurally resemble PilE, modulate pilus functions: DNA uptake, bacterial aggregation, adhesion to human endothelial cells and induction of host cell signalling events^4^.

Two key endothelial cell receptors for type IV pili are CD147 and the β2-adrenergic receptor (β_2_AR). *N. meningitidis* interacts first with CD147, a member of the immunoglobulin (Ig) superfamily, and both PilE and PilV are essential for this event^9^. Inhibiting this interaction prevents the primary attachment of bacteria to both peripheral and brain human endothelial cells *in vitro* and the colonization of human skin and brain vessels *in vivo*^9^. Meningococci then promote signalling events in host endothelial cells by specifically activating the β_2_AR^10^. In contrast to the activation of β_2_AR by cognate agonists, which stimulates heterotrimeric G proteins and G-protein-independent signalling pathways^11^, *N. meningitidis* only induces a biased activation of β_2_AR, leading to the activation of scaffolding proteins known as β-arrestins^10^. β-arrestins then favour the local recruitment and activation of both cytoskeleton-associated and signalling proteins, resulting in remodelling of the apical plasma membrane beneath bacterial colonies^12^,^13^. Ezrin and moesin, which accumulate within 2–4 min after bacterial contact^14^, are key players in the reorganization of the cortical actin cytoskeleton at bacterial adhesion sites^13^. Ultimately, local signalling events result in the formation of membrane protrusions surrounding bacteria and allow them to resist haemodynamic forces^15^. The intimate interaction of meningococcus with endothelial cells, which is essential for vascular colonization, is a pre-requisite for vascular dysfunction^16^.

Despite our understanding of how meningococci interact with endothelial cells and hijack host cell signalling pathways to infect tissues, some key issues remain to be resolved. The stable adhesion of meningococci to vascular cells involves the simultaneous interaction of type IV pili with two independent receptors. Thus, a still unknown mechanism of coordination and/or crosstalk between these two receptors likely exists. Moreover, the relatively low affinity of pilin monomers for CD147 is overcome by their multimeric assembly into type IV pili, which enhances avidity^9^. However, how host cell receptors are organized to provide sufficient binding sites for bacterial ligands remains unknown. Here we report that CD147 and β_2_ARs form hetero-oligomeric complexes, allowing for β_2_AR activation immediately after the initial interaction of bacterial pili with CD147. We also demonstrate that the scaffolding protein α-actinin-4 (Actn4) directly binds to the cytosolic tail of CD147 and organizes the formation of highly ordered clusters of hetero-oligomeric receptor complexes, thereby providing optimal binding strength for meningococcal adhesion to endothelia under shear stress.

## Results

### CD147 and the β2AR receptor form hetero-oligomeric complexes

**Figure 1:**
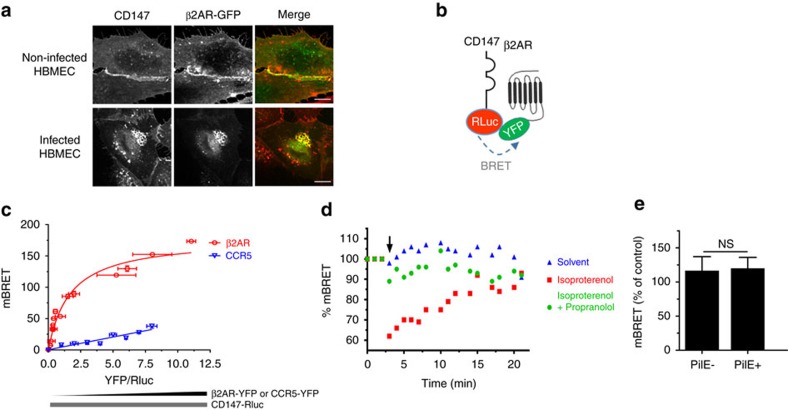
CD147 and the β_2_-adrenergic receptor form hetero-oligomers. (**a**) CD147 and the β_2_AR colocalize at endothelial cell junctions and at sites of bacterial adhesion. HBMECs, transiently transfected with β_2_AR-YFP, were non-infected (top) or infected with meningococci for 2 h (bottom). CD147 (red) and β_2_AR-YFP (green) receptor localization was analysed by confocal microscopy (scale bars, 10 μm). (**b**) Constructs used for BRET assays. CD147 fused to *Renilla* luciferase (CD147-Rluc) and β_2_AR fused to YFP (β_2_AR-YFP) were used as energy donor and acceptor, respectively. (**c**) BRET analysis of CD147–β_2_AR interaction. HEK293 cells were transfected with CD147-Rluc (2 ng) and increasing amounts (0–400 ng) of β_2_AR-YFP or CCR5-YFP. At 24 h after transfection, coelenterazine h (Rluc substrate) was added to measure the BRET signal. Results correspond to one experiment (mean±s.e.m., *n*=3) representative of five independent experiments. (**d**) Isoproterenol modified the BRET_50_. HEK293 cells were transfected with 2 ng CD147-Rluc and 20 ng β_2_AR-YFP, so that energy transfer was close to the BRET50 value. Coelenterazine h (Rluc substrate) was added to measure the basal energy transfer, then 10 μM isoproterenol alone or in combination with 10 μM propranolol, or solvent alone used as control, were added to cells and the energy transfer was measured every 2 min for 20 min. Arrow indicates time of injection of the different ligands. Results correspond to one experiment (mean±s.e.m., *n*=3) representative of four independent experiments. (**e**) The meningococcal pilin PilE does not modify the BRET_50_. HEK293 cells were transfected with 2 ng CD147-Rluc and 20 ng β_2_AR-YFP, so that energy transfer was close to the BRET50 value. Coelenterazine h was added to measure the basal energy transfer, then purified meningococcal PilE was added and the transfer was measured every 2 min for 30 min. Results correspond to one experiment (mean±s.e.m., *n*=3) representative of four independent experiments.

Because CD147-dependent adhesion and β_2_AR activation occur consecutively and in a short time frame, we investigated whether these receptors might be associated in a complex independently of meningococcal infection. The relative cellular distribution of endogenous CD147 and β_2_AR fused to the yellow fluorescent protein (β_2_AR-YFP) was analysed in human bone marrow endothelial cells (HBMECs)^17^. In non-infected cells, CD147 and β_2_AR were present at the luminal surface and enriched at intercellular junctions, where they colocalized (Fig. 1a). On infection by *N. meningitidis*, both receptors massively accumulated beneath bacterial colonies (Fig. 1a), forming typical honeycomb-shaped structures surrounding bacteria, as described previously^9^,^10^.

The proximity of these two receptors was analysed in living cells using bioluminescence resonance energy transfer (BRET)^18^. A fusion protein of CD147 and *Renilla* luciferase (the BRET donor, CD147-Rluc) was expressed in HEK293 cells in the presence of increasing concentrations of β_2_AR-YFP (YFP being the BRET acceptor) (Fig. 1b). A hyperbolic BRET saturation curve was obtained (Fig. 1c), indicating specific basal proximity (≤10 nm) between these two receptors. In a control experiment with the chemokine G-protein-coupled receptor CCR5, only a linear bystander BRET signal was obtained (Fig. 1c). The BRET signal between CD147 and β_2_AR was reduced in the presence of the membrane-impermeable β_2_AR agonist isoproterenol (10 μM), which only binds to surface receptors (Fig. 1d). This effect was inhibited by propranolol, a selective β_2_AR antagonist. Since isoproterenol activation changes β_2_AR conformation^19^, the observed BRET variation likely reflects a change in the relative position of CD147 to β_2_AR in the complex. These data are consistent with a model in which these two receptors form pre-existing complexes at the cell surface.

### CD147–β2AR complex formation facilitates β2AR activation

**Figure 2:**
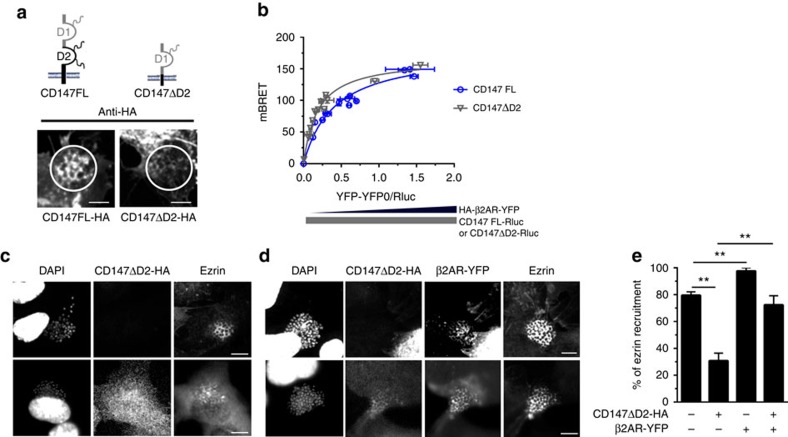
Truncated CD147ΔD2 interferes with CD147–β_2_AR interaction and prevents β_2_AR activation by meningococci. (**a**) CD147ΔD2 does not bind meningococci. HBMECs, transfected with HA-tagged full-length form of CD147 (CD147FL-HA) or a truncated form of CD147 deleted of the proximal extracellular Ig domain (CD147ΔD2), were infected with meningococci for 2 h. Receptor localization was analysed by confocal microscopy using anti-HA antibody. Images are representative of three independent experiments (scale bars, 5 μm). (**b**) CD147ΔD2 efficiently interacts with β_2_AR. HEK293 cells were co-transfected with CD147-Rluc or CD147ΔD2-Rluc (2 and 5 ng, respectively, so that Rluc signal was similar for the two constructs) and increasing amounts of β_2_AR-YFP (0–400 ng) and BRET signal was measured 24 h after transfection. Results correspond to one experiment (mean±s.e.m., *n*=3) representative of three independent experiments. (**c**–**e**) CD147ΔD2 prevents the activation of β_2_AR by meningococci. HBMECs were transfected with CD147ΔD2-HA (**c**) in the absence or (**d**) in the presence of β_2_AR-YFP and infected with meningococci for 2 h and processed for immunofluorescence staining with 4,6-diamidino-2-phenylindole (DAPI) (to visualize bacteria and cell nuclei), anti-HA and anti-ezrin antibodies before analysis by confocal microscopy. Images are representative of six independent experiments (scale bars, 10 μm). (**e**) The ezrin recruitment index was calculated by determining the proportion of colonies that efficiently recruit ezrin at sites of bacterial adhesion (mean±s.e.m., *n*=4 experiments, **P<0.01; one-way analysis of variance (ANOVA)).

Addition of meningococci, or purified PilE or PilV pilins, did not modulate the energy transfer between CD147 and β_2_AR, suggesting that their binding to CD147 does not affect the basal interaction between these receptors (Fig. 1e, shown for PilE). Since the initial adhesion of meningococci to CD147 is independent of β_2_AR^9^ but a prerequisite for β_2_AR activation^10^, we investigated whether CD147–β_2_AR proximity can affect meningococcus-promoted signalling events in host cells. A truncated form of CD147 (CD147ΔD2), with a deletion of the proximal extracellular Ig domain, necessary for the interaction with meningococcal type IV pili^9^, was expressed in cells that also express endogenous CD147. CD147ΔD2 was not recruited to sites of bacterial adhesion, consistent with its inability to interact with meningococci (Fig. 2a). However, CD147ΔD2 remained in close proximity to the β_2_AR, as shown by BRET experiments (Fig. 2b), indicating that it is likely capable of competing with wild-type endogenous CD147 for the association with β_2_AR. Cell signalling in response to meningococcal infection was then compared in endothelial cells expressing exogenous CD147ΔD2 and non-transfected controls (Supplementary Fig. 1). In controls, meningococcal adhesion led to a β_2_AR-dependent recruitment of ezrin at bacterial adhesion sites, as reported^10^. In contrast, ezrin recruitment was markedly impaired in cells expressing exogenous CD147ΔD2, as shown by the more diffuse cytosolic expression of ezrin (Fig. 2c, lower panels) in comparison with control cells (Fig. 2c, upper panels) and a global reduction of ezrin recruitment (Fig. 2e) under bacterial colonies. This effect was rescued by coexpressing exogenous β_2_AR-YFP (Fig. 2d,e), which most likely restores the cellular density of complexes containing full-length CD147 and β_2_AR. Taken together, these results indicate that CD147 and β_2_AR heterotypic complexes control β_2_AR activation and downstream signal pathways on bacterial adhesion to endothelial cells, therefore accelerating and facilitating the adhesion-signalling sequence.

### α-Actn4 associates with CD147–β_2_AR complexes

**Figure 3:**
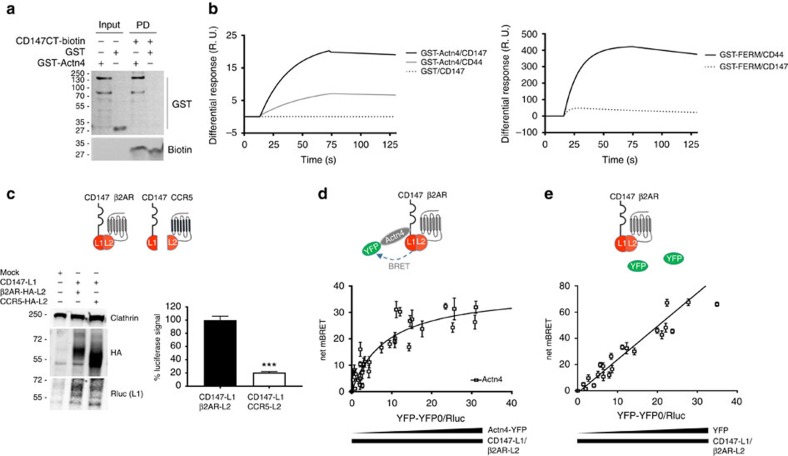
α-Actn4 associates with CD147–β_2_AR complexes. (**a** and **b**) Purified Actn4 directly binds to the cytosolic domain of CD147. (**a**) Pull-down (PD) assay: purified GST–Actn4 fusion protein or GST alone was incubated with biotinylated peptides comprising the cytosolic tail of CD147 (CD147-CT) immobilized on streptavidin beads. Precipitated samples were successively immunoblotted with anti-GST (top) and anti-biotin antibodies (bottom). Input GST proteins were loaded as a control (right lanes). The uncropped blots are shown in Supplementary Fig. 11. (**b**) Surface plasmon resonance analysis of the interaction between Actn4 and the cytosolic domain of CD147 in non-reducing buffer conditions. Left panel: Sensorgrams showing binding of GST–Actn4 fusion protein or GST alone (200 nM) to the cytosolic tail of CD147 or of CD44 as a control. Right panel: Control sensorgrams showing binding of the ezrin FERM domain in fusion with GST to the cytosolic tail of CD147 and CD44. (**c**–**e**) CODA-RET characterization of Actn4 interaction with CD147–β2AR complexes. (**c**) Constructs used for the CODA-RET assays: CD147 was fused to the N-terminal fragment of *Renilla* luciferase (CD147-L1) and the β_2_AR to the C-terminal fragment (β_2_AR-L2). As a control, CCR5 was also fused to the C-terminal fragment (CCR5-L2). (Left panels) The coexpression of the different constructs was confirmed by western blot analysis with anti-HA and anti-Rluc (L1) antibodies. The uncropped blots are shown in Supplementary Fig 12. (Right panel) Luciferase assays: HEK293 cells were co-transfected with varying ratios of CD147-L1 and β_2_AR-L2 or CCR5-L2 and 24 h after transfection coelenterazine h was added to measure the luciferase activity. Optimal results were obtained for a CD147: β2AR ratio equivalent to 9:1. Results correspond to the mean±s.e.m. of *n*=4 independent experiments. (**d** and **e**) CODA-RET assays: Actn4 was fused to YFP at its N terminus. HEK293 cells were co-transfected with CD147-L1 (50 ng), β_2_AR-L2 (450 ng) and increasing amounts (0–400 ng) of Actn4-YFP or YFP. At 24 h after transfection, coelenterazine h (Rluc substrate) was added to measure the BRET signal. The results correspond to the mean (±s.e.m.) of four independent experiments.

We next searched for additional partners that might facilitate CD147–β_2_AR proximity and/or cooperate with CD147–β_2_AR complexes for signalling during meningococcal infection. Proteins co-immunoprecipitated with CD147 from non-infected and infected endothelial cell lysates were analysed using nano-liquid chromatography tandem-mass spectrometry (MS) (Supplementary Fig. 2). Among the 24 identified hits (Table 1), 14 were actin cytoskeleton-associated proteins, such as Actn4, an actin filament crosslinking and bundling protein of the spectrin family, which functions as a molecular scaffold^20^. Functional Actn4 is an antiparallel dimer^21^ that serves as a docking platform for transmembrane adhesion molecules such as intercellular adhesion molecule-1 or integrin β-subunit^22^,^23^ and enhances leukocyte–endothelial cell interactions or traction forces between cells and the extracellular matrix^24^,^25^. We examined whether Actn4 would interact with the cytosolic tail of CD147 using a pull-down experiment between a recombinant glutathione-*S*-transferase (GST)–Actn4 fusion protein and a biotinylated peptide encompassing the 36-residue cytosolic tail of CD147. GST–Actn4 did bind to the peptide immobilized on streptavidin beads, whereas control GST did not (Fig. 3a). Furthermore, surface plasmon resonance (SPR) experiments showed that GST–Actn4 specifically binds to the CD147 peptide immobilized on a sensor chip (Fig. 3b). When performed in a non-reducing buffer that preserves the dimeric/oligomeric form of the molecules (Supplementary Fig. 3A), the affinity of this interaction, assessed from association and dissociation kinetics, was in the nanomolar range (dissociation constant (*K*_d_)=4.00±0.01 nM). Control GST did not interact with the immobilized peptide (Fig. 3b). Additional controls showed that the GST-coupled four-point-one, ezrin, radixin, moesin (FERM) domain of ezrin poorly interacted with the CD147 peptide, whereas this domain interacts strongly (*K*_d_=4.3±0.1 nM) with the immobilized juxta-membrane cytosolic portion of CD44 (Fig. 3b), which contains a known ezrin binding motif^26^. Finally, Actn4 selectivity for CD147 was shown by its much lower affinity for CD44 (*K*_d_=159±43 nM) (Fig. 3b). In a reducing buffer that favours the transition of Actn4 dimers into monomers (Supplementary Fig. 3A), Actn4 still specifically interacted with CD147 in a dose-dependent manner, although the apparent affinity of this interaction was reduced to the micromolar range (*K*_d_=44.6±32.5 μM) (Supplementary Fig. 3B). These data indicate that Actn4 can directly interact with the cytosolic tail of CD147 and suggest that the avidity of this interaction is largely increased by Acnt4 dimerization.

Variation of Actn4 expression in HEK293 cells had no effect on the BRET signal between CD147 and the β_2_AR, indicating that Actn4 is not required for their basal proximity (Supplementary Fig. 4). To address whether Actn4 could interact with preassociated CD147–β_2_AR complexes, we used complemented donor–acceptor resonance energy transfer (CODA-RET), an approach combining bimolecular complementation and BRET^27^,^28^. For this purpose, each receptor was fused at its C terminus to complementary halves of *Renilla* luciferase (L1 and L2), resulting in receptor fusion constructs (CD147-L1 and β_2_AR-L2) that can reconstitute functional luciferase when coexpressed, because of their proximity (Fig. 3c). In a control experiment, 10-fold less luciferase activity was detected when coexpressing CD147-L1 with CCR5-L2 (the chemokine receptor 5 fused to L2) at similar levels, as assessed by western blot (Fig. 3c). BRET experiments were then performed in cells coexpressing CD147-L1 and β_2_AR-L2 in the presence of increasing concentrations of Actn4-YFP, as the BRET acceptor. A hyperbolic BRET saturation curve was obtained (Fig. 3d), indicating specific proximity between these two receptors and Actn4. In contrast, only a linear bystander BRET signal was obtained with YFP alone (Fig. 3e). These data are consistent with a model in which Actn4 associates with CD147–β_2_AR pre-existing complexes.

### α-Actn4 is recruited to bacterial adhesion sites

**Figure 4:**
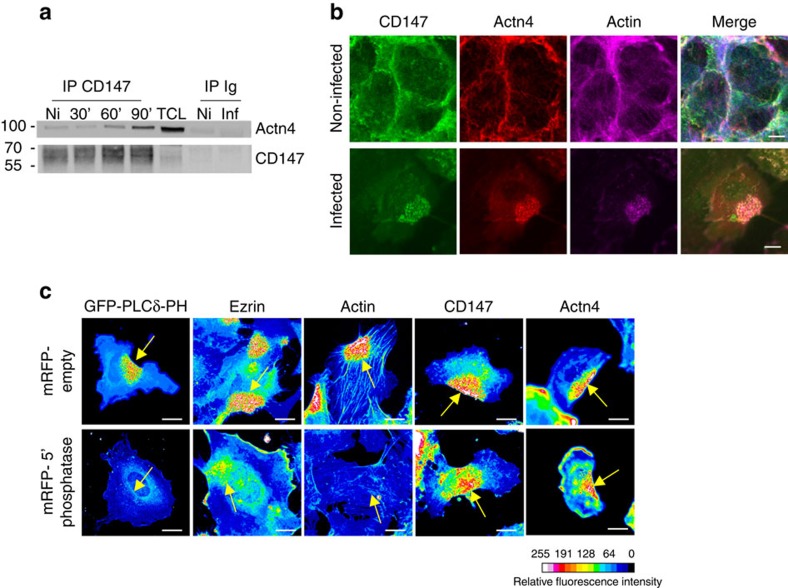
α-Actn4 is recruited to bacterial adhesion sites together with CD147. (**a**) Actn4 co-immunoprecipitates with CD147. HBMECs were either non-infected (Ni) or infected with meningococci for the indicated period of time before lysis. CD147 was immunoprecipitated (irrelevant antibody (Ig) as control) and the obtained samples were successively immunoblotted with anti-Actn4 (top) and anti-CD147 antibodies (bottom). The total cell lysate (TCL) was loaded as a control. (**b**) CD147 and Actn4 colocalize at endothelial cell junctions and at sites of bacterial adhesion. HBMECs non-infected (top) or infected for 2 h with meningococci (bottom) were stained for CD147 (green), Actn4 (red) and actin (blue) and analysed by confocal microscopy. Merged images of the same fields are presented on the right (scale bar, 10 μm). (**c**) Actn4 accumulates at sites of bacterial adhesion independently of PI(4,5)P_2_ production and cortical actin polymerization. HBMECs were transfected with GFP-tagged PH domain of PLC-δ1, along with the membrane-targeted FRB-CFP and the mRFP-FKBP domain constructs (with or without 5′-phosphatase domain as indicated), infected with meningococci for 2 h in the presence of rapamycin to induce the translocation of 5′-phosphatase to the plasma membrane and stained for ezrin, actin, CD147 and Actn4. The colour bar indicates the pixel intensity of normalized fluorescence images (in arbitrary units). Arrows point at sites of bacterial adhesion. Control GFP, mRFP-FKBP, mRFP-FKBP-5-phosphatase and FRB-CFP fluorescence images are shown in Supplementary Fig. 5. Images are representative of five independent experiments (scale bars, 10 μm).

Actn4 co-immunoprecipitated with CD147 from both non-infected and infected endothelial cell lysates, but the association between these proteins markedly increased over time after *N. meningitidis* infection (Fig. 4a). Consistently, CD147 and Actn4 were both enriched and colocalized at cell–cell junctions in non-infected endothelial cells, whereas meningococcal infection induced their massive recruitment to bacterial adhesion sites, where they colocalized with cortical actin (Fig. 4b). It was reported that Actn4 distribution to the plasma membrane is dynamically controlled by phosphatidylinositol-4,5-bisphosphate (PI(4,5)P_2_), cortical F-actin and transmembrane receptors^29^. The recruitment of Actn4 to sites of bacterial adhesion was then examined in cells expressing a membrane-targeted rapamycin-inducible enzymatic system to dephosphorylate PI(4,5)P_2_ (ref. 30) This system efficiently inhibited the production of PI(4,5)P_2_ under bacterial colonies, as assessed by the absence of accumulation of the green fluorescent protein (GFP)-tagged PH domain of PLC-δ1, which binds to PI(4,5)P_2_ with high affinity (Fig. 4c and Supplementary Fig. 5). Although this inhibition prevented both the recruitment of the PI(4,5)P_2_-binding protein ezrin and actin polymerization, both CD147 and Actn4 remained enriched under meningococcal colonies. These observations indicate that the recruitment of Actn4 to bacterial adhesion sites is independent of PI(4,5)P_2_ production and of cortical actin polymerization, and might occur via direct interaction with the cytosolic tail of CD147.

### α-Actn4 governs the organization of CD147–β_2_AR complexes

**Figure 5:**
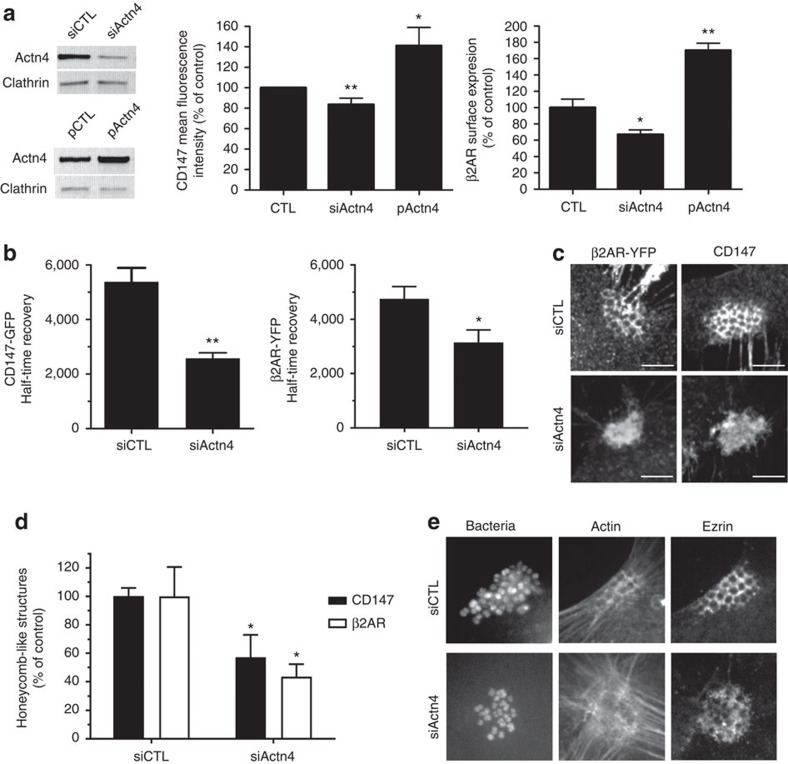
α-Actn4 governs the distribution and dynamics of CD147–β_2_AR complexes at the cell plasma membrane. (**a**) Actn4 modulates the expression of CD147 and β_2_AR at the cell plasma membrane. HBMECs were transfected with siRNA targeting Actn4 (siActn4), control siRNA (siCTL), plasmid encoding Actn4 (pActn4) or empty vector as a control (pCTL). Actn4 expression was addressed by western blot analysis, using clathrin as a control (left panels), and surface expression of endogenous CD147 was determined by FACS analysis (middle panel). In separate experiments, HBMECs were co-transfected with the same siRNA or plasmids together with a HA-β_2_AR-YFP construct, to address surface expression of β_2_AR using anti-HA antibody in relation to the number of YFP-expressing cells (left panel). Histograms represent the mean fluorescence intensity in relation to the control conditions. Representative FACS plots of these experiments are shown in Supplementary Fig. 6. Mean±s.e.m., *n*=3; *P=0.05, **P=0.01, two-tailed Student's *t*-test. (**b**) Actn4 depletion increases the mobility of CD147 and β_2_AR at the cell plasma membrane. HBMECs, co-transfected with CD147-GFP or β_2_AR-YFP and siRNA targeting Actn4 (siActn4) or control siRNA (siCTL), were subjected to FRAP assay. Shown are (left) the halftime recovery of CD147-GFP in *n*=18–22 cells per condition and (right) the halftime recovery of β_2_AR-YFP in *n*=12–22 cells per condition from four independent experiments. Representative fluorescence images of the FRAP experiments and mean FRAP recovery curves are shown in Supplementary Fig. 7. (**c** and **d**) Actn4 depletion disrupts the organized assembly of CD147 and β_2_AR at bacterial adhesion sites. (**c**) Confocal analysis of endogenous CD147 or β2AR-YFP in control (siCTRL) or Actn-4-depleted (siActn4) HBMECs infected for 1 h by *N. meningitidis* (scale bars, 10 μm). (**d**) Percentage of bacterial colonies associated with organized CD147 or β_2_AR assemblies in honey comb-like structures at sites of meningococcal adhesion. Mean±s.e.m.; *n*=3 experiments; *P<0.05, one-way analysis of variance (ANOVA). (**e**) Actn4 depletion disrupts the organized assembly of ezrin and cortical actin polymerization at bacterial adhesion sites. Confocal analysis of ezrin and actin in control (siCTRL) or Actn4-depleted (siActn4) HBMECs infected for 1 h by *N. meningitidis* (scale bars, 10 μm).

We next investigated whether Actn4 might control the distribution and dynamics of CD147–β_2_AR complexes at the plasma membrane. A 90% reduction of Actn4 expression in HBMECs decreased the surface expression of CD147 by 15%, whereas a twofold increase of Actn4 expression increased cell surface CD147 by 40% (Fig. 5a and Supplementary Fig. 6A), showing that Actn4 contributes to stabilize the surface expression of endogenous CD147. Similar results were observed on cell surface expression of an HA-β_2_AR-YFP construct, when coexpressed in Actn4-depleted or -overexpressing cells (Fig. 5a and Supplementary Fig. 6B). The analysis of CD147 dynamics by fluorescence recovery after photobleaching (FRAP) showed a twofold increase in CD147-GFP mobility in Actn4-depleted cells (halftime recovery: 2,520±690 ms, versus 5,380±1,720 ms in control cells) (Fig. 5b and Supplementary Fig. 7). Similarly, an increase in β_2_AR-YFP mobility was observed in Actn4-depleted cells (halftime recovery: 3,150±1,208 ms, versus 4,750±1,886 ms in control cells) (Fig. 5b), indicating that interaction of Actn4 delays lateral diffusion of both CD147 and β_2_AR, probably by promoting interaction with cytoskeletal components.

The distribution of CD147 and β_2_AR in control and Actn4-depleted HBMECs was then examined in the context of meningococcal infection. In control non-depleted cells, endogenous CD147 and exogenous β_2_AR-GFP were massively recruited under bacterial colonies, forming typical ‘honeycomb-like' structures. In Actn4-depleted cells, both CD147 and β_2_AR accumulated at bacterial adhesion sites, but typical honeycomb-like structures were absent in 50% of infected cells (Fig. 5c,d). The organization of the underlying actin cytoskeleton and of ezrin at bacterial adhesion sites was also affected, as expected (Fig. 5e).

**Figure 6:**
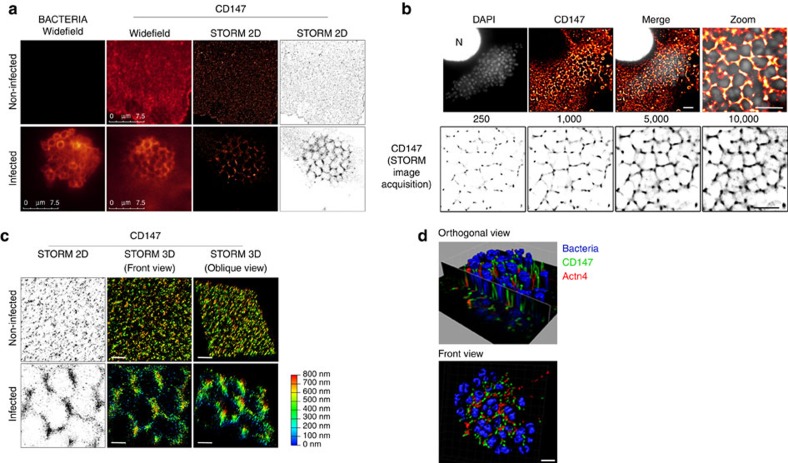
α-Actn4 governs the spatial organization of CD147–β_2_AR complexes at bacterial adhesion sites. (**a** and **b**) CD147 organizes in dense clusters surrounding adherent bacteria. (**a**) HBMECs cells were non-infected or infected for 1 h with meningococci, stained for bacteria and CD147, and analysed by widefield and STORM microscopy on a Leica SR GSD. Fifty thousand frames were recorded for each acquisition and reconstructed using the Leica Application Suite (LAS X) core module. (**b**) HBMECs cells infected for 1 h with meningococci were stained for CD147 and 4,6-diamidino-2-phenylindole (DAPI) to visualize bacteria and cell nuclei (N); imaging was performed on a iMIC imaging work station. Ten thousand frames were recorded and reconstructed using QuickPALM 1.1 ImageJ plug (scale bar, 5 μm). Bottom: Typical STORM reconstruction sequence; legend indicates the number of acquired images used for reconstruction. (**c**) Comparative 2D- and 3D-STORM analysis of CD147 distribution at bacterial adhesion sites. HBMECs cells were infected for 1 h with meningococci and stained for CD147. Images were acquired as in (**a**), on a Leica SR GSD. Shown are the 2D- and 3D-STORM reconstructions of CD147 clusters assembled at bacterial adhesion sites using the Leica LAS X 3D Visualization (scale bars, 1 μm) and the depth colour code (0–800 nm). (**d**) 3D-SIM analysis of CD147 distribution at bacterial adhesion sites. 3D-SIM reconstruction of CD147 and Actn4 clusters at bacterial adhesion sites (scale bar, 2 μm).

The spatial organization of CD147 was further analysed at the molecular level using a photo-switchable antibody coupled to an optical super-resolution (∼20 nm) microscopy technique based on single-molecule localization (dSTORM)^31^. In control HBMECs, CD147 molecules were diffusely expressed at the cell surface of non-infected endothelial cells, whereas these molecules were mostly organized in small molecular complexes surrounding adherent bacteria in infected cells (Fig. 6a,b and Supplementary Movie 1). Images were analysed with SR-Tesseler software to perform a precise and automatic segmentation and quantification of protein organization^32^. This further demonstrated the organization of CD147 in clusters (mean size: 37,700±2,715 nm^2^), whereas only few clusters were detected in non-infected cells (Supplementary Fig. 8). Similar results were obtained using different fluorophores, STORM imaging buffers or image reconstruction systems (Supplementary Fig. 9A–C,E). Furthermore, this cluster organization was not observed for receptors that do not accumulate at bacterial adhesion sites, such as CD71 (Supplementary Fig. 9D), demonstrating the specific organization of these clusters for receptors recruited at bacterial adhesion sites. The small variation in CD147 cluster sizes, together with their regular organization (Fig. 6b), indicated a tightly regulated assembly process. Three-dimensional (3D) reconstruction of dSTORM images (Fig. 6c and Supplementary Movie 2) showed that CD147 clusters were organized along membrane protrusions, surrounding bacteria. This specific organization was confirmed in 3D super-resolution images using 3D-structured illumination microscopy (3D-SIM), which enhances approximately eightfold the spatial resolution of wide-field fluorescence microscopy^33^. Adherent bacteria appeared embedded in long membrane protrusions enriched in CD147 receptors (Fig. 6d).

**Figure 7:**
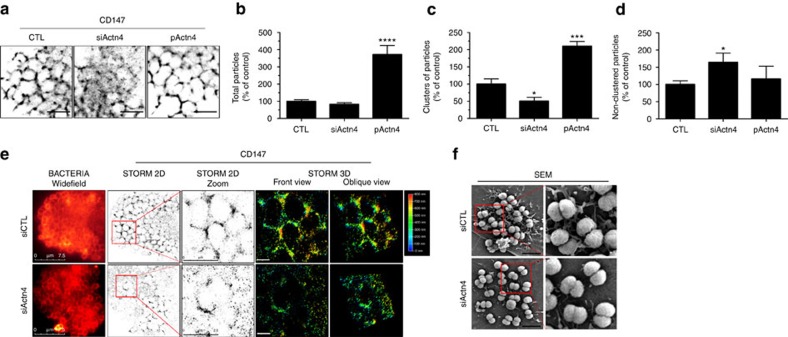
α-Actn4 governs CD147 spatial organization and formation of membrane protrusions at bacterial adhesion sites. (**a**–**d**) Actn4 expression governs the organization of CD147 in clusters at sites of bacterial adhesion. (**a**) STORM reconstruction of CD147 distribution at bacterial adhesion sites in control (CTL), Actn4-depleted (siActn4) or Acnt4-overexpressing (pActn4) HBMEC cells, acquired on an iMIC imaging work station. Ten thousand frames were recorded and reconstructed using QuickPALM 1.1 ImageJ plug (scale bar, 5 μm). (**b**) Ratio of blinking CD147 receptors accumulated per 100 μm^2^ area at bacterial adhesion sites in relation to the control condition (mean±s.e.m., *n*=8, ****P<0.0001, two-tailed Student's *t*-test). (**c**) Ratio of CD147 clusters in 100 μm^2^ at bacterial adhesion sites in relation to the control condition (mean±s.e.m., *n*=8, *P<0.05, ***P<0.001, two-tailed Student's *t*-test). (**d**) Ratio of non-clustered blinking CD147 receptors per 100 μm^2^ at bacterial adhesion sites in relation to the control condition (mean±s.e.m., *n*=8, *P<0.05, two-tailed Student's *t*-test). Quantification as described in the Method section. (**e**) Actn4 depletion alters CD147 spatial distribution at bacterial adhesion sites. HBMECs transfected with siRNA targeting Actn4 (siActn4) or control siRNA (siCTL) were infected for 1 h with meningococci and stained for CD147 and Bacteria. Shown are the bacteria acquired in widefield, the 2D- and 3D-STORM reconstructions of CD147 clusters at bacterial adhesion sites, imaged on a Leica SR GSD and the depth colour code (0–800 nm). Fifty thousand frames were recorded for each acquisition and reconstructed using the Leica Application Suite (LAS X) core module (scale bar, 1 μm). (**f**) Acnt4 depletion alters the formation of membrane protrusions at bacterial adhesion sites. HBMECs transfected with siRNA targeting Actn4 (siActn4) or control siRNA (siCTL) were infected for 1 h with meningococci and analysed by scanning electron microscopy (SEM). Shown are representative scanning electron micrographs of infected control and Actn4-depleted HBMECs (original magnification × 10,000) (scale bar, 5 μm).

In Actn4-depleted cells, the well-structured organization of CD147 around adherent bacteria was markedly affected (Fig. 7a and Supplementary Movie 3). Although the overall density of blinking molecules accumulating at sites of bacterial adhesion was not significantly altered (Fig. 7b), their organization in clusters around adhering bacteria was decreased by 50% compared to control non-depleted cells (Fig. 7c), and the density of blinking molecules sparsely distributed at bacterial adhesion sites increased by 50% (Fig. 7d). Conversely, Actn4 overexpression in HBMECs caused a threefold increase in the overall density of blinking molecules at bacterial adhesion sites compared to control cells (Fig. 7b). These receptors were organized in clusters, as in control cells (Fig. 7a). These data indicate that the assembly of CD147 clusters at bacterial adhesion sites requires a minimal level of Actn4. Moreover, consistent with the correlation between CD147 clusters and the formation of membrane protrusions, CD147 assembled poorly in membrane protrusions of Actn4-depleted cells (Fig. 7e and Supplementary Movie 4). Scanning electron microscopy analysis confirmed the defect in membrane protrusion formation at sites of bacterial adhesion in Actn4-depleted cells compared to control cells (Fig. 7f).

Overall, these data indicate that Actn4 is a key scaffolding molecule for the organization of highly ordered complexes of meningococcal receptors at bacterial adhesion sites, providing the structural backbone for the organization of a 3D honeycomb lattice surrounding the adherent bacteria.

### α-Actn4 determines the strength of bacterial adhesion

**Figure 8:**
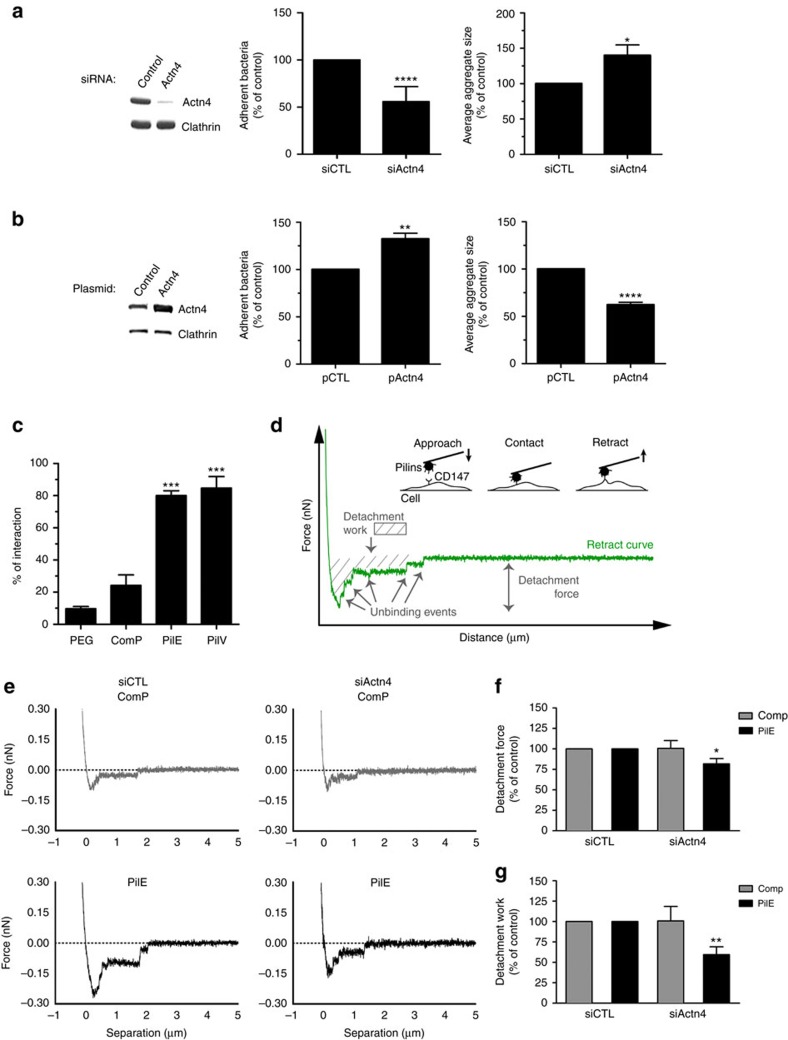
α-Actn4 regulates the strength of meningococcal adhesion to human endothelial cells. (**a** and **b**) Actn4 expression modulates bacterial adhesion to human endothelial cells under shear stress. HBMECs were transfected with (**a**) control (CTL) or Actn4 siRNA or with (**b**) control (CTL) or Actn4 plasmid. Right panels: Western blot analysis of Actn4 levels, using clathrin as a control. Middle panels: Number of adhesion events (meningococci adhering individually or in aggregates) following a 10 min infection under shear stress (0.04 dynes cm^−2^). Left panels: Average size (in pixels) of the meningococcal colony. The number and the size of the adherent individual bacteria and/or bacterial aggregates were quantified using the ImageJ software. Representative images of the adherent bacteria in each condition are shown in Supplementary Fig. 10. Mean±s.e.m., *n*=4, ****P<0.0001, **P<0.01, *P<0.05, two-tailed Student's *t*-test. (**c**) The meningococcal pilin PilE and PilV selectively interact with the endothelial cell surface. AFM analysis of retraction forces between cantilever tips functionalized with PEG linker alone (control) or with PEG linker plus MBP-pilins (ComP, PilE or PilV) and the apical membrane of living HBMECs. Bar plots indicate the percentage of force curves with unbinding events. Mean±s.e.m.; *n*=7 experiments (448 force curves and 7 cells per condition), ***P<0.001, one-way analysis of variance (ANOVA). (**d**–**g**) Actn4 expression regulates interaction strength between meningococcal pilins and their endothelial receptors. (**d**) Schematic representation of the different parameters measured. The detachment force corresponds to the total rupture force of adhesive bonds involved during interaction of pilin-coated bead with the cell surface throughout a series of unbinding events, as measured by the jumps on the force–distance retraction curve. Detachment work represents the ‘work of deadhesion' that contains both mechanical components inherent to the cell type and adhesive elements due to pilin–receptor complexes that break on retraction of the bead. (**e**) AFM analysis of the interaction between 2-μm-sized beads coated with ComP or PilE and living Actn4-depleted or control HBMECs. Shown are the (**f**) detachment forces and the (**g**) detachment work measured. Mean±s.e.m.; *n*≥6 experiments (270 force curves and 30 cells per condition), *P<0.05, **P<0.01, one-way ANOVA.

Given the central role of Actn4 in CD147–β2AR receptor clustering and in the formation of cellular protrusions on meningococcal infection, we investigated if Actn4 levels would modulate meningococcal adhesion to human endothelial cells. HBMECs were grown in controlled laminar flow chambers to quantitatively analyse early bacterial contacts with the endothelial cell surface under physiological conditions of shear stress^2^. A 95% reduced expression of Actn4 in HBMECs was associated with a 50% decrease in meningococcal adhesion (Fig. 8a). In control conditions, bacteria mostly adhered as diplococci or small aggregates, whereas in Actn4-depleted cells meningococci mainly adhered as large aggregates (Fig. 8a and Supplementary Fig. 10A). Conversely, Actn4 overexpression in HBMECs increased bacterial adhesion events, with a higher percentage of single diplococci or small-size aggregates (Fig. 8b and Supplementary Fig. 10A). In control experiments, ezrin overexpression was not associated with any noticeable change in adhesion events (Supplementary Fig. 10B).

These data suggest that Actn4-dependent formation of CD147–β2AR clusters provides a sufficient density of bacterial adhesion points (avidity), thereby compensating for the weak interaction between single receptor complexes and bacterial pilins. To test this hypothesis, atomic force microscopy (AFM) experiments were conducted to measure the binding strength of the meningococcal pilins to the endothelial cell surface. AFM tips coated with purified meningococcal PilE, PilV or control ComP pilins, fused to the maltose-binding protein, were used to measure binding forces. Non-coated tips were used as a control. Curves depicting the percentage of retraction between the pilin-coated pyramidal tips and the cell surface, and unbinding events were compared (percentage of interaction). As expected, the percentage of interaction events observed with non-coated tips and ComP-coated tips did not differ significantly, whereas the number of events was increased by eightfold with PilE- or PilV-coated tips (Fig. 8c). Similar interaction forces with endothelial cells (26.6±0.6 and 28.8±0.6 pN, respectively) were measured for PilE or PilV. These data indicate a specific interaction of these two pilins with the surface of endothelial cells, consistent with their known interaction with CD147 and β2AR^9^,^10^. The interaction of PilE with control and Actn4-depleted HBMECs was then compared, using 2 μm ball cantilevers, to mimic the size of the bacteria for interaction measurements (Fig. 8d). These cantilevers were coated with purified PilE or ComP, as a control (Fig. 8e). As observed with tip cantilevers, no specific interaction was observed with ball cantilevers coated with ComP on either control or Actn4-depleted cells (Fig. 8e–g). However, with PilE-coated ball cantilevers, detachment forces of adhesive bonds (Fig. 8e,f) and the detachment work (Fig. 8e,g) were significantly reduced by 20% and 40%, respectively, in Actn4-depleted cells compared to control cells. The interaction strength between meningococcal pilins PilE and endothelial cell surface receptors is thus reduced on Actn4 depletion.

Overall, these data demonstrate that the organization of CD147–β_2_AR hetero-oligomeric complexes in highly ordered clusters via Actn4 scaffolding is critical for the interaction of meningococci with human endothelial cells, by enhancing the strength of the interaction and for promoting specific signalling events.

## Discussion

In this study, we show that CD147 and β_2_AR are organized in pre-existing complexes at the surface of endothelial cells and that a previously unappreciated cytoskeletal protein partner, Actn4, scaffolds the assembly of highly ordered clusters of receptor complexes in response to bacterial adhesion. This multimolecular assembly process ensures sufficient binding strength to the initial interaction of meningococcal type IV with CD147 and then the rapid subsequent activation of the β_2_AR, which ultimately reinforces bacterial adhesion by eliciting multiple signalling events. This amplification mechanism allows bacteria to adhere to vascular walls *in vivo* and resist haemodynamic forces of blood flow.

The functional hetero-oligomerization of the β_2_AR with a non-G-protein-coupled receptor (GPCR) family member was not reported previously, although there are several reports of functional interaction between GPCR and receptors belonging to other families or integral membrane proteins, such as CD4, ion channels, RAMP, RTP, MRAP and MHC molecules^34^,^35^. Here we demonstrated that CD147, β_2_AR and Actn4 form stable pre-existing molecular complexes, which are exploited by meningococci for adhesion, signalling and infection. CD147 and β_2_AR can form independently functional homo- and hetero-oligomers^19^,^36^. CD147 can function as a chaperone for other transmembrane proteins, such as monocarboxylate transporters, promoting their polarized targeting to the basolateral membrane^37^. β_2_AR homo-oligomerization contributes to receptor trafficking to the plasma membrane^38^, whereas hetero-dimers between the β_2_AR and the other β_2_AR subtypes, β_1_AR and β_3_AR, were reported to drive specific signalling cascades, pharmacological properties and trafficking behaviour^39^,^40^. Resonance energy transfer approaches have emerged as tools of choice to monitor protein proximity in living cells, in particular to study the formation of homo- and heterocomplexes between GPCR family members^19^ or between GPCR and non-GPCR family members^41^. The observation of an energy transfer between CD147-Rluc and β_2_AR-YFP indicates molecular proximity of the BRET donor and acceptor of 10 nm or less, consistent with their hetero-oligomerization. The molecular proximity between these two receptors was further confirmed by luciferase complementation. These approaches, however, do not provide any direct information about the stoichiometric arrangement of these receptors and cannot thus discriminate between 1:1 hetero-dimerization and association of homodimers. Moreover, it cannot rule out the hypothesis that the proximity of CD147 and of β_2_AR results from the independent interaction of these proteins with a common scaffolding partner. However, the observation that the β-adrenergic agonist isoproterenol modulates the energy transfer between CD147-Rluc and β_2_AR-GFP in non-infected cells demonstrates that these receptors are preassembled at the plasma membrane, independently of meningococcal infection. Preassociation of the early adhesion and signalling receptors represents a clear advantage for the bacteria in the context of tissue infection. The proximity of CD147 and β_2_AR contributes to the spatial and temporal facilitation of adrenergic receptor activation. Unlike β_2_AR natural agonists, which bind to a pocket formed by the seven transmembrane domains of the receptor, the meningococcal ligands PilE and PilV likely interact with the N-terminal region of the β_2_AR^10^. The same bacterial ligands interact with the extracellular region of CD147, although via a different binding interface. Indeed, mutation of the C-terminal domain of PilE altered the activation of the β_2_AR without affecting the adhesion to human dermal endothelial cells^42^. Based on the observation that type IV pili can transit into an elongated conformation that exposes hidden epitopes previously buried in the pilus fibre^43^,^44^,^45^, a plausible model of β_2_AR activation would imply the formation of a ternary complex between type IV pili and the two cellular receptors. Alternatively, bridging the host cell receptors might facilitate the transactivation of the β_2_AR by CD147, either by direct interaction or via signalling or scaffolding molecular partners. Similar examples of utilization of physiological hetero-dimeric receptor complexes by pathogens have already been described, one of the most well studied being the complex between CD4 and the chemokine receptors CCR5 or CXCR4, which are the coreceptors for the human immunodeficiency virus^46^,^47^.

Following adhesion, meningococci promote the organized assembly of CD147–β_2_AR clusters at sites of bacterial adhesion. We found that the formation of these clusters requires sufficient concentration of a third partner, Actn4, indicating the necessity of an optimal stoichiometric ratio of receptors and of this cytoskeletal protein. These clusters provide molecular platforms for the elongation of membrane protrusions that were shown to rapidly surround bacteria after their initial adhesion^14^ and to maintain stable adhesion of growing colonies despite the forces exerted by the blood flow^2^,^15^. In this context, the functional role of these structures would be to provide a sufficient number of binding sites for polymeric bacterial ligands.

Although originally discovered as an actin-linking protein, Actn4 regulates a variety of cell functions, including cell adhesion and contractility, cell migration, cytokinesis, vesicular trafficking, tight junction assembly or transcriptional regulation^20^. Actn4 is also a docking platform for transmembrane adhesion molecules, such as intercellular adhesion molecule-1 or the β-subunit of integrins^22^,^23^, contributing to enhanced leukocyte–endothelial cell interactions or traction forces between cells and the extracellular matrix, respectively^24^,^25^,^48^,^49^. Actn4 can create a connected network with actin filaments to modulate cellular dynamics and force generation^50^. Here, we demonstrated the existence of a direct binding between the cytosolic domain of CD147 and Actn4. Furthermore, using CODA-RET, we demonstrated the ability of Actn4 to associate with preformed CD147–β_2_AR complexes. Actn4 controls the surface expression, the dynamics, as well as the assembly of CD147–β_2_AR complexes at the endothelial cell surface, most likely by providing docking sites for the cytosolic domain of CD147. The organized assembly of these receptors in clusters increases the overall binding strength of meningococci to endothelial cells. Additionally, Actn4 functions as a signalling node essential for coordinating bacterial attachment and cytoskeleton dynamics leading to membrane protrusion formation. Actn4 might therefore also scaffold signalling and/or regulatory molecules under bacterial colonies contributing to the elongation of these membrane protrusions. This finding demonstrates how the highly specific organization of cellular receptors impacts a host–pathogen interaction and the subsequent triggering of signalling events.

In addition to its role in targeting the monocarboxylate transporters to the plasma membrane^37^, CD147 has been previously shown to functionally interact with other transmembrane proteins such as the adherens junction-associated protein 1 (Shrew-1)^51^. We found here that CD147 co-immunoprecipitated several other cytoskeleton-associated proteins in addition to Actn4, such as Actn1 and filamin A and B, two proteins known to connect membrane receptors to the cytoskeleton. Despite their high homology, Actn1 and Actn4 are not functionally redundant and display different roles^52^,^53^,^54^. However, we cannot rule out the possibility that these proteins collaborate with Actn4 to finely tune the structural organization of CD147–β_2_AR clusters.

In conclusion, we have unraveled the mechanism of cooperation of two endothelial receptors from different families, in the context of infection by a bacterial pathogen, and identified a third protein partner that orchestrates their 3D rearrangement early after bacterial adhesion. The cooperative nature of the interaction between CD147 and β_2_AR receptors and their molecular assembly by Actn4 constitutes a new paradigm for the generation of high-affinity docking sites for an invasive bacterial pathogen.

## Methods

### Antibodies and reagents

Anti-hCD147 monoclonal antibody (mAb) (clone MEM-M6/1) was purchased from AbD Serotec, anti-hCD44 mAb (clone J173) from Immunotech, anti-HA mAb (clone 12CA5) from Roche, anti-clathrin mAb from Transduction Laboratories and rabbit anti-Actn4 from Abcam. Polyclonal antisera raised against ezrin and GST were obtained from Pr Paul Mangeat (CRBM, Montpellier, France) and Dr Franck Perez (Institut Curie, Paris, France), respectively. mAb raised against PilE (20D9) and polyclonal antiserum raised against meningococcal 2C4.3 strain were described previously^10^. Mouse IgG isotype control from Santa Cruz (sc-2025) was used as a control. Secondary antibodies used for immunofluorescence labelling and immunoblotting were from Jackson ImmunoResearch Laboratories. For STORM analysis, anti-mouse Ig conjugated with Alexa Fluor 647 and Alexa Fluor 555 were purchased from Life Technologies. 4,6-Diamidino-2-phenylindole, rhodamine-phalloidin and isoproterenol were purchased from Sigma-Aldrich. Biotinylated peptides were purchased from ProteoGenix. The antibody dilutions/concentrations used in this work are provided in Supplementary Table 1.

### Plasmids

Plasmids encoding β_2_AR-YFP or fused to both HA and YFP (HA-β_2_AR-YFP) and CCR5 fused to YFP were described previously^19^. Plasmids encoding human ezrin, human α-Actn4, human CD147, PLC-δ1–PH-GFP and the rapamycin-inducible PI(4,5)P_2_ dephosphorylation constructs were kindly provided by Dr Monique Arpin (Institut Curie), Dr Alain Duperrey (Institut Albert Bonniot, Grenoble, France), Dr Mickael Bukrinsky (The George Washington University, Washington, DC, USA), Dr Georges Bismuth (Institut Cochin, Paris, France) and Dr Tamas Balla (National Institute of Child Health and Human Development, Bethesda, MD, USA), respectively. The HA tag was introduced by PCR into pcDNA3-CD147 vector using the Phusion Site-Directed Mutagenesis Kit (Finnzyme) and deletion of the CD147 D1 domain was generated by PCR on the previous construction. The YFP tag was introduced by PCR into pcDNA3-α-Actn4 vector. The pcDNA3.1 vectors encoding full-length Rluc8 or fragments for the L1 (residues 1–229) or L2 (residues 230–311) were kindly provided by Dr Jonathan A. Javitch (Colombia University, New York, NY, USA); HA-tagged-CD147 and -β_2_AR were introduced in frame in these vectors by PCR. All the constructs were confirmed by sequencing analysis. The primer sequences used in this work are provided in Supplementary Table 2.

### Bacterial strain and meningococcal pilins

*N. meninigitidis* Nm2C4.3 strain (formerly clone 12) is a piliated capsulated Opa^−^ Opc^−^ variant of the serogroup C meningococcal clinical isolate 8013 (ref. 55) cultured as described previously^56^. Briefly, before experiments, bacteria were grown overnight on gonococcal base agar solid medium (4% GC medium base; Difco), 1% agar, 0.4% glucose, 0.2 mg ml^−1^ thiamine, 0.0005% Fe(NO_3_)39H_2_O and 0.01% L-glutamine at 37 °C under 5% CO_2_. Several colonies were then selected and grown in DME supplemented with 0.1% bovine serum albumin (BSA) for 2 h and were finally diluted to 10^7^ bacteria per ml suspension for cell infection. Fragments of PilE, PilV and ComP lacking the 28 N-terminal amino acids of the mature proteins fused to the maltose-binding protein (MBP) were produced in *E. coli* and purified on amylose resin (New England Biolabs), as described previously^10^.

### Cell culture, transfection and infection

HBMECs, a human bone marrow capillary endothelial cell line provided by Dr B. Weksler^17^, were cultured and infected as described previously^13^. Briefly, cells were grown in DMEM glucose 4.5 g l^−1^+GlutaMax (Life Technologies) supplemented with 10% heat-inactivated fetal bovine serum, 7.5 μg ml^−1^ endothelial cell growth supplement (Sigma-Aldrich), 7 UI heparin (Sigma-Aldrich) and 10 mM HEPES. Bacteria grown on gonococcal base agar agar plates were adjusted to *A*_600_=0.02 in prewarmed DMEM GlutaMax medium containing 0.1% BSA and incubated for 1 h 30 min at 37 °C. The bacterial culture was then diluted to 10^7^ bacteria per ml. Confluent monolayers of HBMECs were then overlaid for 30 min with the bacterial suspension (multiplicity of infection=100). Cells were washed three times with the DMEM/BSA medium to remove non-adherent bacteria and infection was allowed to proceed for 2 to 3 h.

To silence the expression of Actn4, the following short interfering RNA (siRNA) 5′-GCAGCAGCGCAAGACCUUC55-3′ and 5′-GAAGGUCUUGCGCUGCUGC55-3′ purchased from Eurogentech were used. SiGENOME Non-Targeting siRNA from Thermo Scientific (D-001210-03-05) was used as control siRNA. Knockdown efficiency was assessed by immunoblots and immunofluorescence analysis 48 h after transfection and cells were infected concurrently with Nm2C4.3. HEK293 cells (ATCC; CRL-1573) were maintained in DMEM supplemented with 10% fetal bovine serum and penicillin/streptomycin. siRNA or plasmids were transfected using GeneJuice-Transfection Reagent (Novagen), according to the manufacturer's instructions.

### Adhesion assays under shear stress

Meningococcal adhesion on HBMECs was assayed under shear stress conditions, as described previously^2^. Briefly, after transfection with the indicated siRNA or plasmids, HBMECs were grown on IBIDI chambers for 48 h and then submitted to laminar flow (0.04 dynes cm^−2^) under an inverted microscope. GFP-expressing bacteria were introduced in the flow chamber for 10 min, then the medium was introduced under flow for 10 min to get rid of non-adherent bacteria and fixed. Adhesion of bacteria was recorded using an Olympus CKX41 inverted microscope with a × 40 objective. The total number of adhesion events (that is, GFP-expressing meningococci adhering in single diplococci or in aggregates) and the average size of the bacterial aggregates (measured in μm^2^; one diplococci=3 μm^2^) were quantified using the ImageJ Software.

### Proteomic analysis

Analysis was performed by the 3P5 proteomics facility of the Université Paris Descartes, as follows:

In-gel trypsin digestion: In-gel digestion with trypsin was carried out as described^57^ using a digester/spotter robot (Tecan, Männedorf, Germany) at all steps. Gel bands were destained two times with a mix of 100 mM ammonium bicarbonate (ABC): pure acetonitrile (can; from Carlo Erba) (1:1 volume) for 45 min at 22 °C and then dried using pure ACN for 15 min. Gel bands were then treated with 25 mM ABC containing 20 mM dithiothreitol for 1 h at 60 °C, then alkylated by 55 mM iodoacetamide in 25 mM ABC for 30 min in the dark at 22 °C, washed two times with 25 mM ABC, shrunk two times with pure ACN for 15 min and finally dried out using a vacuum centrifuge (Eppendorf) for 10 min. Gel pieces were preincubated with 13 μl of 12.5 ng μl^−1^ modified trypsin ‘Sequencing Grade' (Promega, Madison, WI, USA) in 40 mM ABC–10% ACN (pH 8.0), for 30 min on ice. Excess liquid was removed and gel pieces were incubated in 20 μl of 25 mM ABC overnight at 37 °C. After digestion, peptides were collected in the supernatant and pooled with two 1% formic acid gel washes and one pure ACN gel wash. Extracts were then dried out using a vacuum centrifuge.

Reverse-phase nanoliquid chromatography fractionation: Dried peptide eluates were redissolved in 20 μl resuspension solution consisting of 0.1% trifuoroacetic acid (TFA; from Fluka) and 10% ACN, vortexed and centrifuged 5 min at 12,000 *g*. A 10 μl portion was injected on an Ultimate 3000 Nano-HPLC (Thermo). Peptides were purified and first concentrated on a C18 PepMap precolumn (0.3 mm ID × 5 mm, 100 Å pore size, 5 μm particle size from Thermo) at a flow rate of 30 μl min^−1^ in 0.1% TFA and 2% ACN (solution A). Subsequently, peptides were separated on a C18 PepMap100 analytical reverse phase column (75 μm ID × 150 mm, 100 Å pore size, 3 μm particle size from Thermo) at 300 nl min^−1^ with a 32 min gradient of solution B (20% solution A mixed vol:vol with 80% ACN) rising from 7% B to 50% B. Fractionation was carried out for each sample using the Probot Automated Fraction Collector (Dionex). Fraction collection was started after a 13 min dead volume delay following the injection signal and was performed directly on a matrix-assisted laser desorption/ionization target (blank plate; ABSciex). Fractions were collected every 10 s for a total of 192 spots per sample. Eluent and matrix solutions were mixed on-target. α-Cyano-4-hydrocinnamic acid (Laserbio-Labs) matrix was dissolved at 2.5 mg ml^−1^ in 70% ACN containing 0.1% TFA

Offline mass spectrometry analysis: Mass spectra were measured with a 4,800 matrix-assisted laser desorption/ionization–time of flight–time of flight mass spectrometer (ABSciex) equipped with a Nd:YAG-pulsed laser (355 nm wavelength, <500 ps pulse and 200 Hz repetition rate). Spectra acquisition and processing were performed using the 4,000 Series Explorer Software (ABSciex) version 3.5.28193 build 1011 in positive reflectron mode at fixed LASER fluency with low mass gate and delayed extraction. External plate calibration was performed using Pepmix1 peptides from ABSciex. For each fraction, steps of 50 spectra in the range of 850 to 4,000 Da were acquired. Five hundred spectra per sample were summed, processed to obtain monoisotopic values from isotopes clusters with a raw spectra signal-to-noise ratio of 20. MS spectra generated by each nLC fraction were treated automatically, allowing the 12 most abundant precursors to be selected for fragmentation starting with the least abundant unless its intensity was higher in a neighbouring fraction. A cutoff was applied at a minimum s/n of 15. Neighbouring precursors within 200 resolutions were excluded. One thousand MS/MS spectra per precursor were summed by increment of 50 and were subjected to baseline subtraction and Savitsky–Golay smoothing (with a polynomial order of 4 and 15 point across peak).

Protein identification: Extracted MS/MS peaklists were subsequently submitted to an in-house mascot (Matrix Science) version 2.2 search engine^58^. The Swiss-prot database (May 2007, 267,354 sequences; 98,158,671 residues) was used restricted to the *Homo sapiens* subset of sequences (16,529 sequences). Parent and fragment mass tolerances were, respectively, set to 100 p.p.m. and 0.3 Da, partial modification (oxidization) of methionines was allowed. Carbamidomethylation of cysteins by iodoacetamide were considered as complete. Missed trypsin cleavage sites were limited to 1. A filter was applied to the search to reduce false positives and matching redundancies of the same peptide in several hits. All peptide matches above 1% risks of random matching were eliminated (*P*<0.01). Individual minimum peptide score was set to 15. False-positives rates were evaluated using Mascot. Under these stringent parameters, the minimum protein score was 34. Unless a specific peptide could differentiate them, the best matching protein was selected even when a subset of peptides could match another protein isoform. The probability score calculated by the software was used as a primary criterion for correct identification.

### BRET and CODA-RET assays

HEK293 cells (2 × 10^5^ per well of a 6-well plates) were transfected with 2 ng plasmid DNA coding for the BRET donor (CD147-Luc, CD147ΔD2-Luc) and increasing amounts of BRET acceptor plasmids (β_2_-YFP, CCR5-YFP; 0–400 ng per well). Twenty-four hours after transfection, cells were resuspended in Hank's balanced salt solution and distributed in 96-well plates (Perkin-Elmer plates; 10^5^ cells per well). After addition of the luciferase substrate, coelenterazine h (5 μM final concentration), luminescence at 485 nm and fluorescence at 530 nm were measured simultaneously in a Mithras LB940 Plate Reader. The BRET ratio was calculated as: ((emission at 530 nm/emission at 485 nm)−(background at 530 nm/background at 485 nm)), where background corresponds to signals in cells expressing the Rluc fusion protein alone under the same experimental conditions. For better readability, results were expressed in milli-BRET units (mBRET), 1 mBRET corresponding to the BRET ratio multiplied by 1000. BRET ratios were plotted as a function of ([YFP-YFP_0_])/(Rluc), where YFP is the fluorescence signal at 530 nm after excitation at 485 nm, and Rluc the signal at 485 nm after addition of coelenterazine h. YFP_0_ and Rluc_0_ correspond to the same values in cells expressing the Rluc fusion protein alone. For the β_2_AR stimulation assays, after reading of the basal energy transfer, 10 μl of vehicle, isoproterenol (agonist) or propranolol (antagonist) at a final concentration of 10 μM was added per well and the energy transfer was measured during 20 min. To address the effect of bacterial adhesion, after reading of the basal energy transfer, bacterial suspension or purified meningococcal pilins MBP-PilE or MBP-PilV (10 μg ml^−1^) were added and the energy transfer was measured during 20 min.

To perform the CODA-RET assays, HEK293 cells (1 × 10^5^ per well of a 12-well plates) were transfected with plasmids DNA coding for the BRET donors (900 ng CD147-L1 and 100 ng β_2_AR-L2) and increasing amounts of BRET acceptor plasmids (Actn4-YFP or YFP alone; 0–400 ng per well). Twenty-four hours after transfection, cells were resuspended in Hank's balanced salt solution and distributed in 96-well plates (Perkin-Elmer plates). After addition of the luciferase substrate, coelenterazine h (5 μM final concentration), luminescence at 485 nm and fluorescence at 530 nm were measured simultaneously in a Mithras LB940 Plate Reader and BRET ratio was calculated and plotted as above.

### Pull-down assays, immunoprecipitations and immunoblotting

For pull-down assays, 1 μg of biotinylated peptide encoding the cytosolic sequence of CD147 (Biotin-AYDKRRKPEDVLDDDDAGSAPLKSSGQHQNDKGKNVRQRNSS) purchased from Proteogenix was immobilized on streptavidin-coated beads for 30 min at 4 °C. After three washes in PBS, coated and control non-coated beads were incubated with GST-Actn4 or GST alone (0.2 μg in PBS, 0.2% BSA) for 1 h at 4 °C. After three washes, beads were lysed with Laemmli buffer and the amount of coated peptides and of co-precipitated GST fusion proteins were assessed by immunoblot analysis.

For the immunoprecipitation experiments, cells were washed once with cold PBS and lysed in 1% *n*-dodecyl β-D-maltoside, 25 mM Tris-HCl (pH 7.5), 140 mM NaCl, 2 mM EDTA, 10 μg ml^−1^ each of aprotinin/leupeptin/pepstatin, 1 mM 4-(2-aminoethyl)benzenesulfonyl fluoride hydrochloride and 1 mM orthovanadate. After centrifugation, the cleared lysates were used for immunoprecipitation with specific antibodies. Precipitated proteins were separated on SDS–PAGE gels and transferred to nitrocellulose (GE Healthcare). After blocking for 1 h in PBS/1% BSA/0.05% Tween-20, filters were probed overnight with specific antibodies and revealed in ECL (Amersham), after probing with peroxidase-coupled secondary antibodies. For proteomic analysis, precipitated proteins were separated on SDS–PAGE gels and stained with Coomassie Blue.

### Flow cytometry

Cell surface labelling with non-conjugated antibodies (anti-CD147, anti-HA) was performed by sequential incubation of 5 × 10^5^ cells with primary antibodies for 30 min at 4 °C in cold FACS buffer (PBS, 3% FCS, 0.01% sodium azide), followed by incubation of the cells with conjugated-secondary antibody for 30 min at 4 °C in cold FACS buffer. Cell surface labelling with fluorescent-conjugated antibodies was performed by incubation of 5 × 10^5^ cells with coupled antibodies for 30 min at 4 °C in cold FACS buffer. After three washes with FACS buffer, cells were fixed with 4% PFA and analysed by flow cytometry (FC500 flow cytometer; Beckman Coulter).

### Fluorescence recovery after photobleaching

HBMECs transiently transfected with a GFP-tagged CD147 construct or a YFP-tagged β_2_AR construct, together with siRNA targeting Actn4 (siActn4) or control siRNA (siCTL), were seeded on 35 mm dishes (Ibidi, Biovalley). Imaging was performed in DMEM medium, 50 mM HEPES and 10% fetal bovine serum on a iMIC imaging work station from TILL Photonics, on the back aperture of a × 100 oil (NA 1.49) objective plus a × 1.5 of internal microscope lens. The regions of interest were photobleached for 25 ms ten times at maximum 491-nm laser power (50 mW). Subsequently, time-lapse images were collected onto a EMCCD camera (iXon Andor Technology) until the bleached signal reached a stable level. FRAP curves from three independent trials with 12 to 22 cells per condition were derived by fitting the normalized fluorescence at each time point versus time into a one-phase association model plugged into the Prism Software. Fmax, which represents the mobile fraction of the molecule in the bleached region, and 1/2, which is the time to recover half of the maximum fluorescence and is inversely correlated to the diffusion coefficient, were derived from this curve.

### Surface plasmon resonance

For SPR experiments, the fusion proteins GST-Actn4, GST alone or the GST-FERM domain of ezrin used as a control were diluted either in a non-reducing (PBS, 0.02% Tween-20) or a reducing (10 mM HEPES, 150 mM NaCl, 1 mM EDTA, 1 mM dithiothreitol, 0.005% Tween-20) running buffer. To control the impact of these buffers on the dimerization status of Actn4, Actn4-GST or GST alone as a control (final concentration 100 nM) were incubated for 30 min with 250 μM freshly prepared solution of bissulfosuccinimidyl suberate crosslinker, then separated on SDS–PAGE gels and transferred to nitrocellulose to analyse their electrophoretic migration by immunoblot. Interaction studies between α-Actn4 and CD147 were performed on a BIAcore T200 Instrument (BIAcore Life Sciences, GE Healthcare Europe). Streptavidin-coated sensor chips (Series S sensor chip SA; GE Healthcare) were used to capture a biotinylated peptide encoding the cytosolic sequence of CD147 (Biotin-AYDKRRKPEDVLDDDDAGSAPLKSSGQHQNDKGKNVRQRNSS) or the juxta-membrane region of CD44 (Biotin-NSRRRCGQKKKLVINSG), purchased from Proteogenix, at a flow rate of 5 μl min^−1^ in the non-reducing or the reducing running buffer. The density was controlled at an increased response level of 630 and 286 response units (RUs), for CD147 and CD44, respectively depending on molecular weight. The fusion proteins GST-Actn4, GST alone or the GST-FERM domain of ezrin diluted in the running buffers were used as analytes and injected at a flow rate of 25 μl min^−1^ for 60 s, followed by a dissociation time of 60 s. Unspecific binding was subtracted from the response using blank runs performed on a surface without immobilized peptide. Output sensorgrams were analysed using the BIAevaluation 4.1.1 Software.

### Confocal immunofluorescence microscopy

HBMECs were grown to confluence on Permanox coverslips (Thermo Fischer Scientific). After the indicated transfection and/or infection, cells were fixed and labelled as described previously^56^. Image acquisitions were performed with a DMI6000 microscope (Leica, × 63). Image analysis were carried out with the ImageJ Software (NIH). Quantitative analysis of protein recruitment under bacterial colonies was determined as the proportion of colonies showing underlying proteins of interest. At least 30 colonies were observed per coverslip. Each experiment was repeated at least three times.

### Stochastic optical reconstruction microscopy

After transfection with the indicated siRNA or plasmids, HBMECs were seeded on 22 mm high-precision cover glasses with thickness of 170 μm (No. 1.5H, Marienfield). Seventy-two hours after transfection, cells were infected with meningococci for 1 h, fixed in 4% paraformaldehyde for 10 min, blocked with 3% BSA/PBS for 10 min, labelled with the anti-hCD147 antibody (1:100 in 1% BSA-PBS) for 1 h, then incubated with goat anti-mouse Alexa Fluor 647F(ab′)2 secondary antibody fragments (Life Technologies; A-21237) (1:1,000) and 4,6-diamidino-2-phenylindole (1:10,000 in 1% BSA-PBS) for 1 h to label both bacterial and endothelial cell nuclei. Labelled preparations were mounted in 50% Vectashield in PBS 1 × pH 7.4 on microscope slides with cavities (Marienfield) and sealed with a twinsil 22 silicon-glue (Rotec) as described previously^59^. For comparison, labelled preparations were imaged in reducing buffer containing (50 mM Tris (pH 8.0), 10 mM NaCl, 10 mM 2-aminoethanethiol (Fluka), 0.5 mg ml^−1^ glucose oxidase (G2133; Sigma-Aldrich), 40 μg ml^−1^ catalase (Sigma-Aldrich) and 20% (w v^−1^) glucose), as described previously^31^,^60^. 2D imaging was performed on an iMIC imaging work station from TILL Photonics, a 640 nm, 100 mW laser (iBeam smart) on the back aperture of a × 100 oil (NA 1.49) objective plus a × 1.5 of internal microscope lens. Fluorescence was imaged onto a EMCCD camera (iXon Andor Technology) with effective pixel size of 106.6 nm. Laser intensity on the sample measured before the objective was ∼60 mW. The 10,000 frames were recorded for each acquisition and reconstructed using QuickPALM 1.1 ImageJ plug or ThunderSTORM with the following settings: Image filtering: B-spline order: 3; B-spline scale: 2.0. Localization of molecules: peak intensity threshold: 2*std(Wave.F1); connectivity: 4-neighbourhood. Subpixel localization of molecules: Method: PSF integrated gaussian, fitting radius 3 px, fitting method; weighted least squares, initial sigma: 1.6 px.

To analyse the organization of the blinking molecules, 2D and 3D STORM imaging was performed on a Leica SR GSD 3D system, with a × 160 oil (NA 1.43) objective. Fluorescence was collected onto an EMCCD camera iXon Andor technology, providing an effective pixel size of 100 nm and processed with the LAS X Software (Leica). Approximately 50,000 frames were recorded for each acquisition with 10 ms exposure time, EM gain of 300 and the number of photons per pixels set to 50. 2D and 3D reconstructions were obtained from stacks (16 planes) with 50 nm axial step size, using the LAS X Software (Leica). The complex organization of CD147 was analysed using the SR-Tesseler Software using default parameters with a density factor=1.3 (ref. 32). Quantifications were performed on seven 2D-reconstruction images (mean cluster size=37,700±2,715 nm^2^). Quantification of the number of CD147 clusters and number of non-clustered blinking CD147 receptors per 100 μm^2^ at bacterial adhesion sites was performed using both SR-Tesseler and ImageJ Software programs.

### Structured illumination microscopy

HBMECs were seeded on Zeiss high–performance coverlips no. 1.5, infected with meningococci for 1 h, fixed in 4% paraformaldehyde for 10 min and labelled as described for STORM. Labelled preparations were mounted in DAKO Fluorescent Mounting Medium (S3023) on StarFrost 76 × 26 mm^2^ microscope slides. SIM images were acquired on a Zeiss ElyraPS1 microscope with × 100 oil-immersion lens (Plan-Apochromat NA 1.46), and a resolution of 100 nm along the *x*–*y*-axis and 300 nm along the *z*-axis (Z-step of 0.116 nm). Super-resolution SIM images were obtained from 15 images (five different phases of three different angular orientations of the illumination pattern), collected on an EMCCD camera (1,004 × 1,002 pixels), processed with Zen (Zeiss) Software and analysed using Imaris 7.7 (Bitplane, Oxford Instruments).

### Atomic force microscopy

Force spectroscopy was performed between pilins and the endothelial cell surface. After transfection with the Actn4 siRNA (along with the appropriated siRNA controls), HBMECs were grown on glass bottom WillCo-dish plates (WillCo Wells B.V.) for 48 h. Purified MBP-pilins were coated either on AFM pyramidal tips (Bruker DNP 0.06 N m^−1^) for specificity measurement on non-depleted cells (Fig. 8c) or on 2 μm SiO_2_ ball cantilevers (sQube ref. CP-PNP-SiO) to mimic the size of the bacteria for interaction measurements (Fig. 8e,f). Cantilevers were cleaned using plasma O_2_, then amino-functionalized and finally covalently coated with pilins using a acetal-PEG-NHS linker^61^. All the experiments were performed at 37 °C on a commercial stand-alone AFM (NanoWizard3; JPK Instruments) combined with an inverted optical microscope (AxioObserver.Z1; Zeiss) driven under the JPK NanoWizard Software 4.3 and Zen Blue 2012. AFM was operated in contact/force curves mode in HBMEC imaging buffer (DMEM glucose 4.5 g l^−1^+Glutamax+HEPES 25 mM). The spring constant was calibrated using the thermal method of the JPK Software for each cantilever. The cantilevers were neither engaged on the edge nor on the soma of the cell.

For the specificity experiments (Fig. 8c), force mapping scans were performed using a force threshold of 1 nN with a 1 s contact delay, a 6 μm Z ramp with closed loop on, on a 10 μm^2^ area, at a retract velocity of 8 μm s^−1^. Force curves were then automatically processed using in-house developed software for the detection of unbinding events. For the interaction experiments (Fig. 8e,f), a force mapping scan was made using a force threshold of 1 nN with a 1 s contact delay, with a 10 μm Z ramp with closed loop on, on a 6 μm^2^ area, at a retract velocity of 5 μm s^−1^. Data were normalized for each experiment so as to eliminate all possible bias due to calibration and coating efficiency, each cantilever scanning both SiCTL and SiActn4 samples with crossed order. This ensured straight comparison between cell populations. Force curves were then automatically processed using in-house developed software for the measurement of detachment forces and works. Plots and sample curves were drawn using GraphPadPrism v.6.

### Scanning electron microscopy analysis

Cells grown on glass coverslips were infected with meningococci for 1 h, fixed with 4% paraformaldehyde for 10 min, treated with 1% osmium tetroxide in water, in the dark for 1 h and dehydrated with increasing ethanol concentration baths before drying with a critical point drier (Quorum Technologies K850, Elexience, France). Dry coverslips were mounted on stubs, coated with 5 nm platinum (Quorum Technologies Q150T, Elexience, France) and observed with a Zeiss SEM Merlin Compact VP (Zeiss, France).

### Statistical analysis

Statistical significance was assessed with the Student's *t*-test and one way analysis of variance by the Prism Software (GraphPad Software).

### Data availability

The mass spectrometry proteomics data have been deposited to the ProteomeXchange Consortium via the PRIDE partner repository with the data set identifier PXD006212. All other data supporting the findings of this study are available within the paper and its Supplementary Information files.

## Additional information

**How to cite this article:** Maïssa, N. *et al*. Strength of *Neisseria meningitidis* binding to endothelial cells requires highly-ordered CD147–β_2_-adrenoceptor clusters assembled by alpha-actinin-4. *Nat. Commun.* **8**, 15764 doi: 10.1038/ncomms15764 (2017).

**Publisher's note:** Springer Nature remains neutral with regard to jurisdictional claims in published maps and institutional affiliations.

## Supplementary Material

Supplementary Information Supplementary Figures and Supplementary Tables

Supplementary Movie 1 CD147 receptors organize as dense clusters at bacterial adhesion sites.2D-STORM reconstruction sequence of CD147 distribution within clusters assembled at bacterial adhesion sites (related to Fig. 6b). Control HBMECs cells infected for 1 hour with meningococci were fixed and stained for CD147 using an Alexa Fluor 647 conjugated secondary antibody to perform optical super-resolution microscopy technique, allowing single-molecule localisation (dSTORM). 10,000 frames were recorded for each acquisition and reconstructed using QuickPALM 1.1 ImageJ plug with the default settings.

Supplementary Movie 2 CD147 receptors are enriched in membrane protrusions in which adherent bacteria are embedded. Typical 3D-STORM reconstruction of CD147 receptor distribution at bacterial adhesion sites, related to Fig. 6c. Control HBMECs cells infected for 1 hour with meningococci were fixed and stained for CD147 using an Alexa Fluor 647 conjugated secondary antibody to perform 3 dimension superresolution microscopy technique (3D-STORM). 50,000 frames were recorded for each acquisition and stacks (16 planes) with 50 nm axial step were reconstructed using LAS X software (Leica).

Supplementary Movie 3 Actn4 depletion alters CD147 clustering at bacterial adhesion sites.Typical 2D-STORM reconstruction sequence of CD147 distribution at bacterial adhesion sites in Acnt4-depleted cells (related to Fig. 7a). Actn4-depleted HBMECs cells infected for 1 hour with meningococci were fixed and stained for CD147 using an Alexa Fluor 647 conjugated secondary antibody to perform optical super-resolution microscopy technique (dSTORM). 10,000 frames were recorded for each acquisition and reconstructed using QuickPALM 1.1 ImageJ plug with the default settings.

Supplementary Movie 4 Actn4 depletion alters the spatial distribution of CD147 at bacterial adhesion sites.Typical 3D-STORM reconstruction of CD147 receptor distribution at bacterial adhesion sites in Actn4 depleted cells, related to Fig. 7e. Actn4-depleted HBMECs cells infected for 1 hour with meningococci were fixed and stained for CD147 using an Alexa Fluor 647 conjugated secondary antibody to perform 3 dimension super-resolution microscopy technique (3D-STORM). 50,000 frames were recorded for each acquisition and stacks (16 planes) with 50 nm axial step were reconstructed using LAS X software (Leica).

## Figures and Tables

**Table 1:**
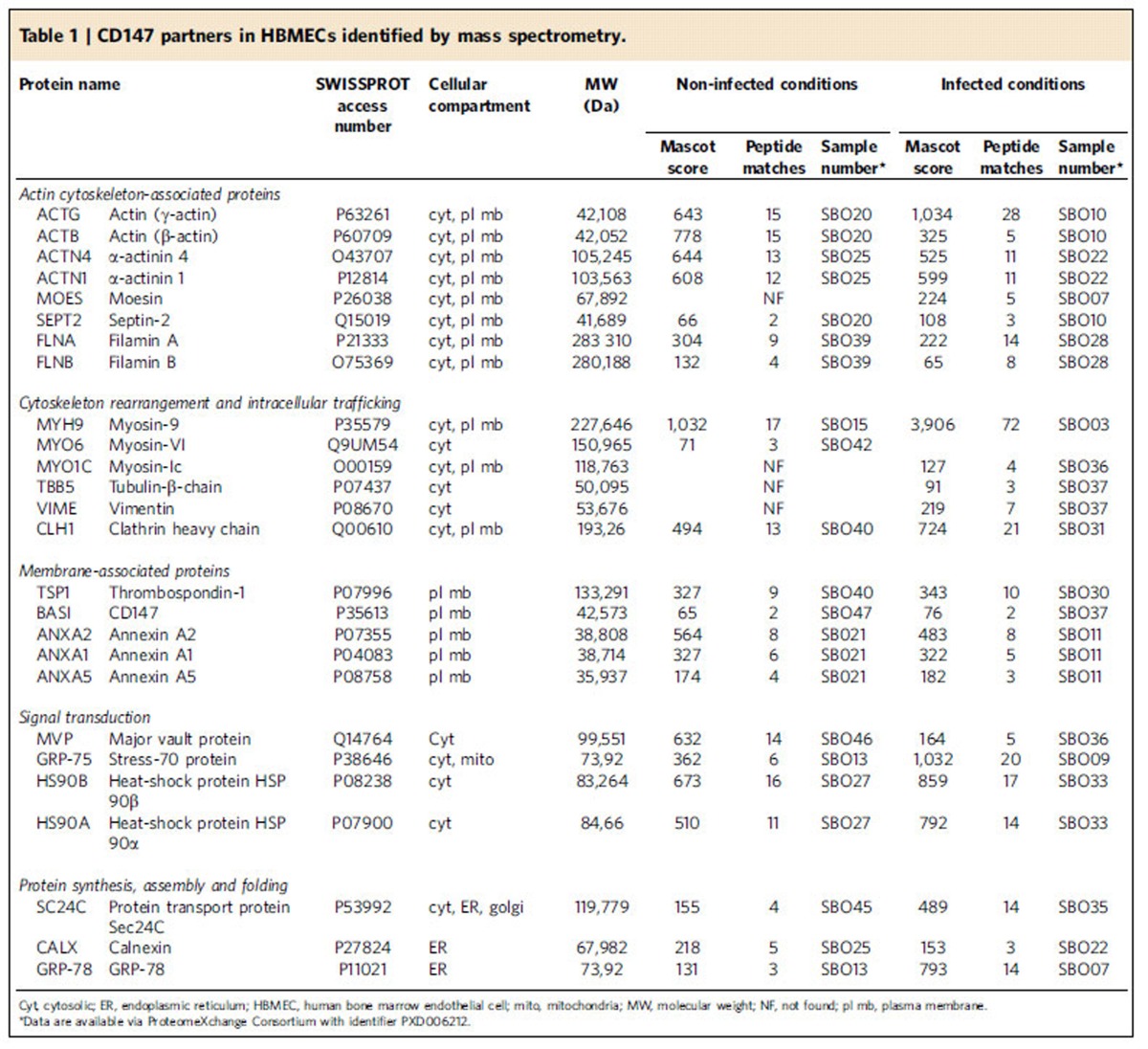
CD147 partners in HBMECs identified by mass spectrometry.
