# Supplementary material for: Strength of Neisseria meningitidis binding to endothelial cells requires highly-ordered CD147/β2-adrenoceptor clusters assembled by alpha-actinin-4
Source: Nat Commun. 2017 Jun 1;8:15764. doi: 10.1038/ncomms15764 (PMC5461506; doi:10.1038/ncomms15764)
Supplement: Supplementary Information — Supplementary Figures and Supplementary Tables [file ncomms15764-s1.pdf]

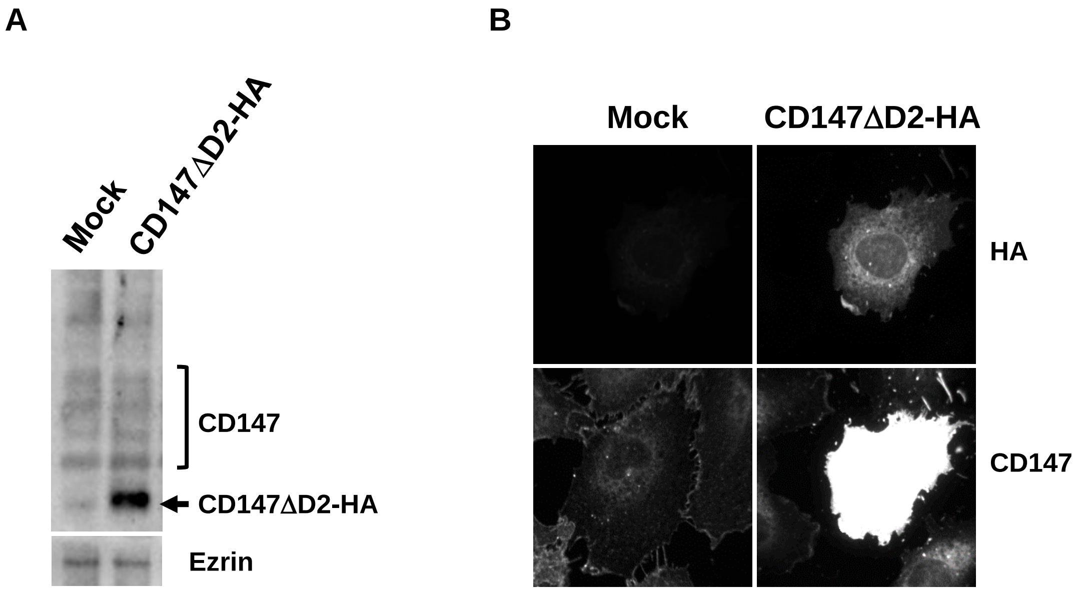

Supplementary Figure 1: Expression analysis of the CD147ΔD2-HA construct.
HBMECs were transiently transfected with CD147ΔD2-HA and expression efficiency was assessed by western blot (A) and immunofluorescence analysis (B) using anti-CD147 and anti-HA antibodies. Expression level of CD147ΔD2-HA was more 10 fold higher than endogenous CD147.

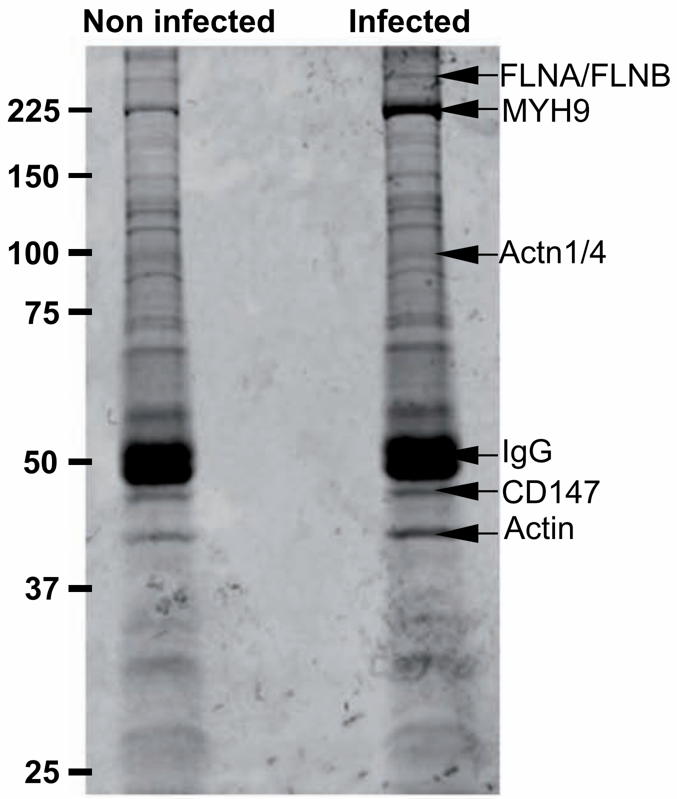

Supplementary Figure 2: Identification of CD147 interacting partners by mass spectrometry.
HBMEC cells were either non-infected or infected by meningococci for 2h before lysis. CD147 was immunoprecipitated, samples were resolved by SDS-PAGE and bands were visualized by coomassie blue staining. Proteins were analyzed by mass spectrometry nano-LC tandem/MS/MS. The list of the identified proteins is reported in table 1.

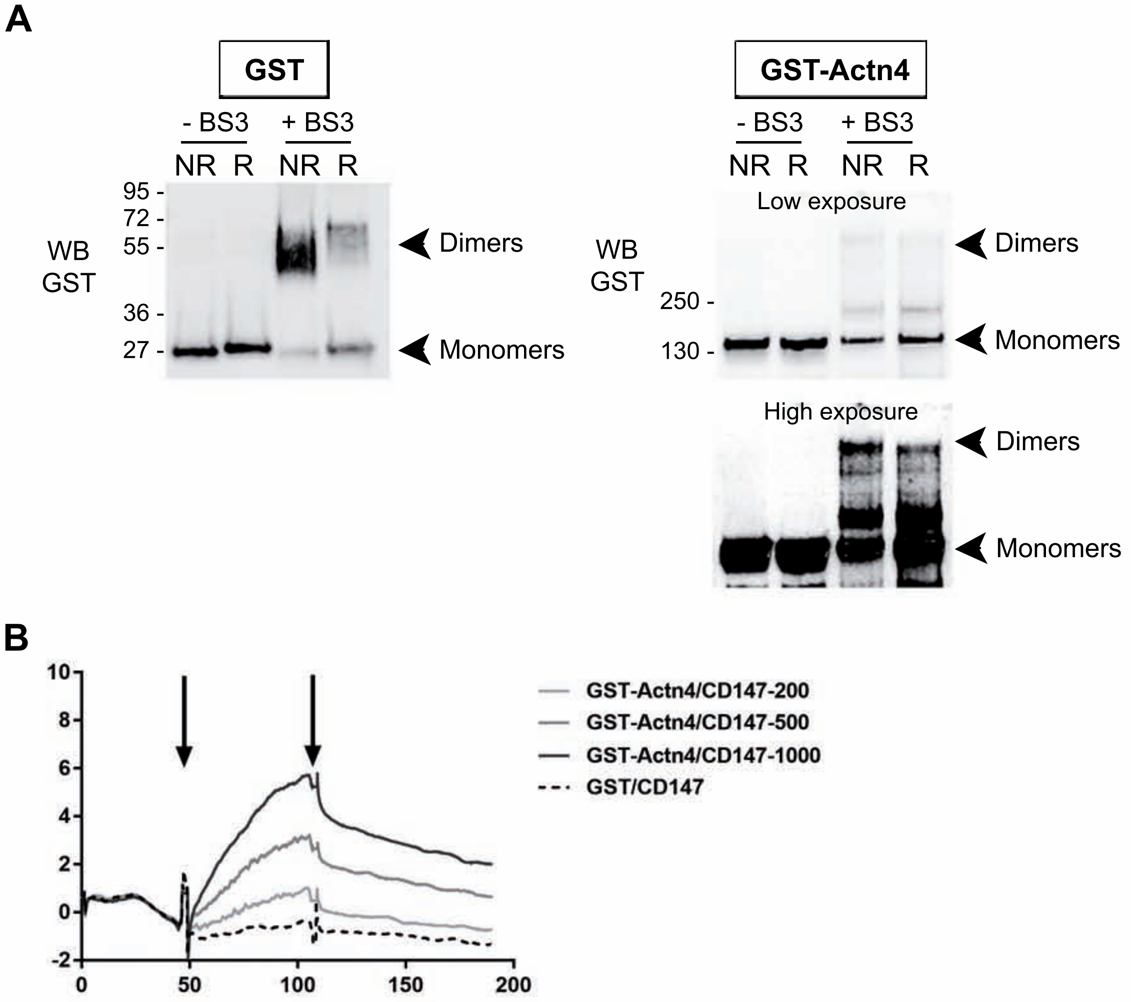

Supplementary Figure 3: Purified Actn4 directly binds to the cytosolic domain of CD147.
(A) Dimerization analysis of the GST fusion proteins by BS3 cross linking: Immunoblots showing the electrophoretic migration of GST (left panel) or GST-Actn4 (right panel) when incubated in a reducing (R) or a non-reducing (NR) buffer in the presence (+) or in the absence (-) of the BS3 crosslinker. Arrows point at monomeric and dimeric forms of the proteins.
(B) Surface plasmon resonance analysis of the interaction between Actn4 and the cytosolic domain of CD147 in reducing buffer conditions: Sensorgrams showing binding of GST-Actn4 fusion protein (200, 500 or 1000 nM) or GST alone (1000 nM), as a control to the cytosolic tail of CD147.

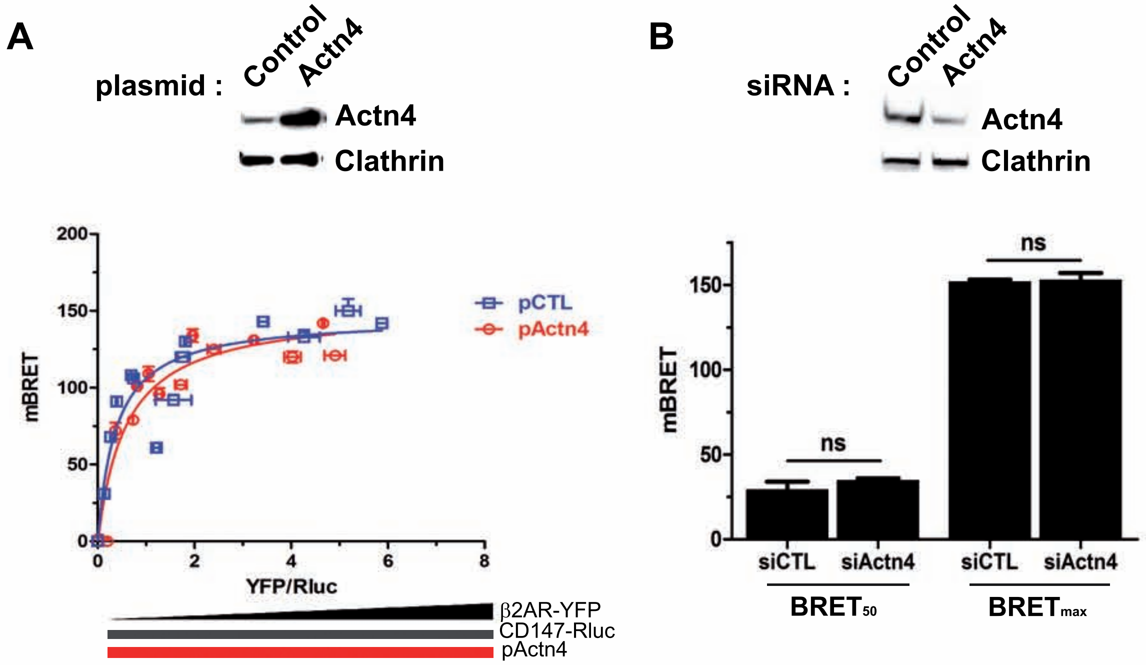

Supplementary Figure 4: Alpha-Actinin4 does not govern the assembly of CD147/β2AR complexes.
(A) BRET analysis of CD147-β2AR interaction in cells overexpressisng Acnt4. HEK-293 cells were co-transfected with control (CTL) or Actn4 plasmids together with CD147-Rluc (2 ng) and increasing amounts of β2AR-YFP (0 to 400 ng). Top panel: western blot analysis of Actn4 overexpression, using clathrin as a control. Lower panel: BRET signal. Results correspond to one experiment (Mean ± sem, n = 3) representative of 3 independent experiments.
(B) BRET analysis of CD147-β2AR interaction in Acnt4 depleted cells. HEK-293 cells were transfected with control (CTL) or Actn4 siRNA, and 48h later, the cells were transfected with 2 ng CD147-Rluc and 20 ng or 400 ng β2AR-YFP so that energy transfers were close to the BRET50 and BRETmax values, respectively. Top panel : western blot analysis of Actn4 depletion, using clathrin as a control. Lower panel: 72h after siRNA transfection, and 24h after plasmid transfection, the BRET signal was measured. Results correspond to two independent experiments (Mean ± sem).

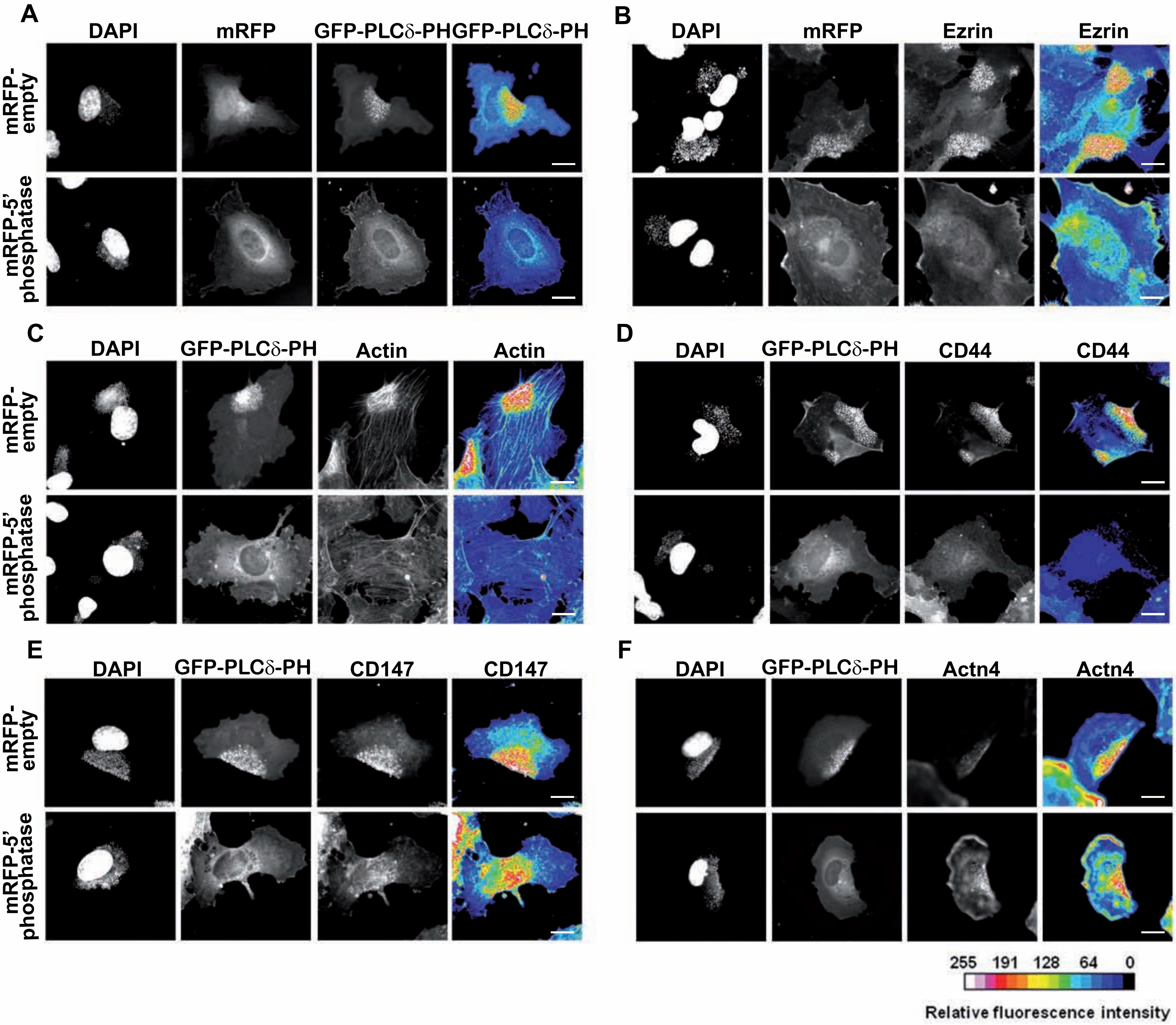

Supplementary Figure 5: PI(4,5)P2 hydrolysis does not affect Acnt4 recruitment to sites of bacterial adhesion.

HBMEC cells were transfected with GFP-tagged PH domain of PLCδ1, along with the membrane-targeted FRB-CFP and the mRFP-FKBP domain constructs (with or without 5'-phosphatase domain as indicated), and were infected with meningococci for 2h in the presence of rapamycin to induce the translocation of 5'-phosphatase to the plasma membrane. Cells were fixed and processed for immunostaining for Ezrin (B), Actin (C), CD44 (D), CD147 (E) and Actn4 (F) together with DAPI to visualize adherent bacteria, and cells expressing mRFP and GFP-PLCγ1-PH were analyzed by fluorescence microscopy. Images are representative of 5 independent experiments (scale bars: 10 μm).

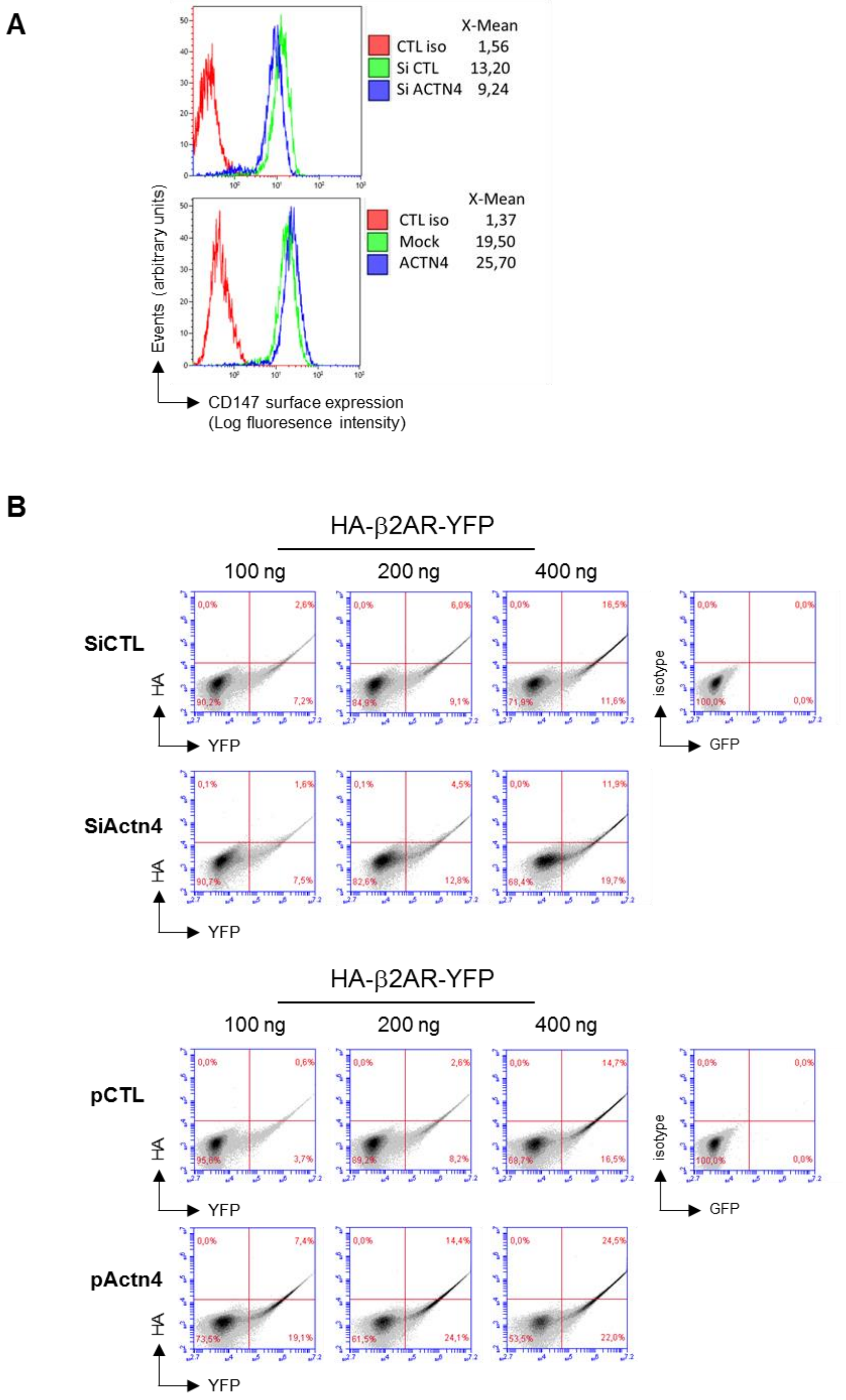

Supplementary Figure 6: Actn4 modulates the expression of CD147 and β_2AR at the cell plasma membrane.

(A) FACS analysis of CD147. HBMEC cells were transfected with siRNA targeting Actn4 (siActn4) or control siRNA (siCTL), (upper panel), or plasmid encoding Actn4 (pActn4) or empty vector as a control (pCTL) (lower panel) and surface expression of endogenous CD147 was determined by FACS analysis. Mean fluorescence intensities are reported. As a control, cells were stained with the secondary antibody alone (red curves).

(**B) FACS analysis of β_2AR**. HBMEC cells were co-transfected with the same siRNA (upper panels) or plasmids (lower panels) together with a HA-β_2AR-YFP construct, to address surface expression of β_2AR using anti-HA antibody in relation to the number of YFP-expressing cells**.** Right panels: as a control, cells were stained with the secondary antibody alone.

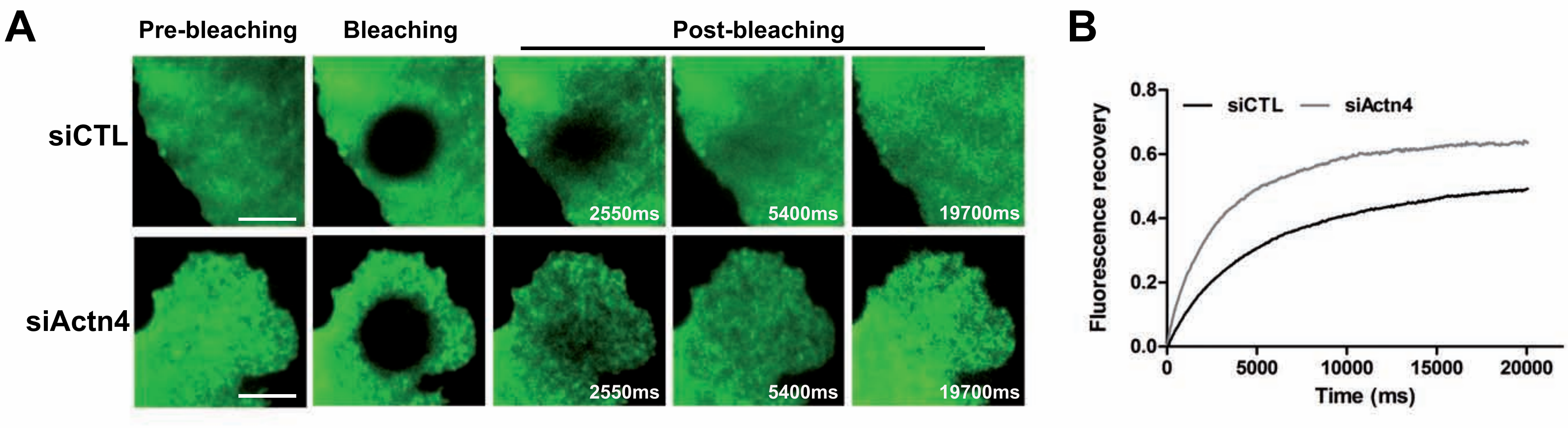

Supplementary Figure 7: Actn4 depletion increases the mobility of CD147.

Fluorescence Recovery After Photobleaching (FRAP) was used to measure the dynamics of CD147-GFP in control and Actn4-depleted HBMEC cells. HBMEC cells were co-transfected with CD147-GFP and siRNA targeting Actn4 (siActn4) or control siRNA (siCTL). 72h after transfection, fluorescence of CD147-GFP was bleached in small areas at the periphery of transfected HBMEC cells. (A) Images obtained before and after photobleaching at the indicated time points (scale bar: 10 µm). (B) Fluorescence recovery rates were measured (shown are mean FRAP recovery curves of CD147–GFP in n=18 to 22 cells per condition from 4 independent experiments) and the halftime of recovery was determined (reported on Figure 5B).

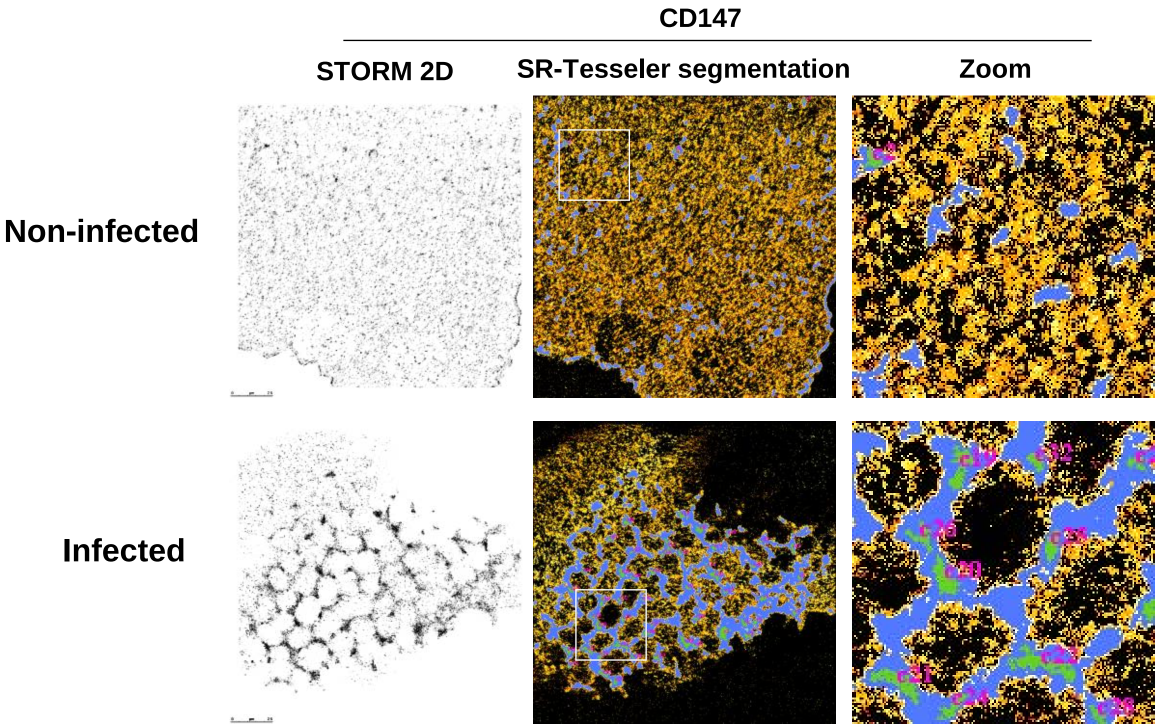

Supplementary Figure 8: SR-Tesseler analysis of CD147 localization.
Shown is representative automatic segmentation and quantification analysis of CD147 localization in non-infected and infected HBMEC cells. (Left) 2D reconstructions of CD147 staining using the Leica Application Suite (LAS X) core module: Original data sets composed of 54359 and 145213 detections in non-infected cells and infected cells, respectively. (Middle) Automatic segmentation performed with SR-Tesseler: CD147 staining contour (blue) and clusters (green). (Right) magnification of the clusters. Numbers correspond to the automatic quantification of the identified clusters. Quantifications were performed on 7 2D-reconstruction images: mean radius of individual clusters = 109 ± 29 nm; mean size = 37700 ± 2715 nm^2; Average local density = 146 ± 12 detections/10.000 nm^2. Only 3 clusters were detected in the non-infected cells.

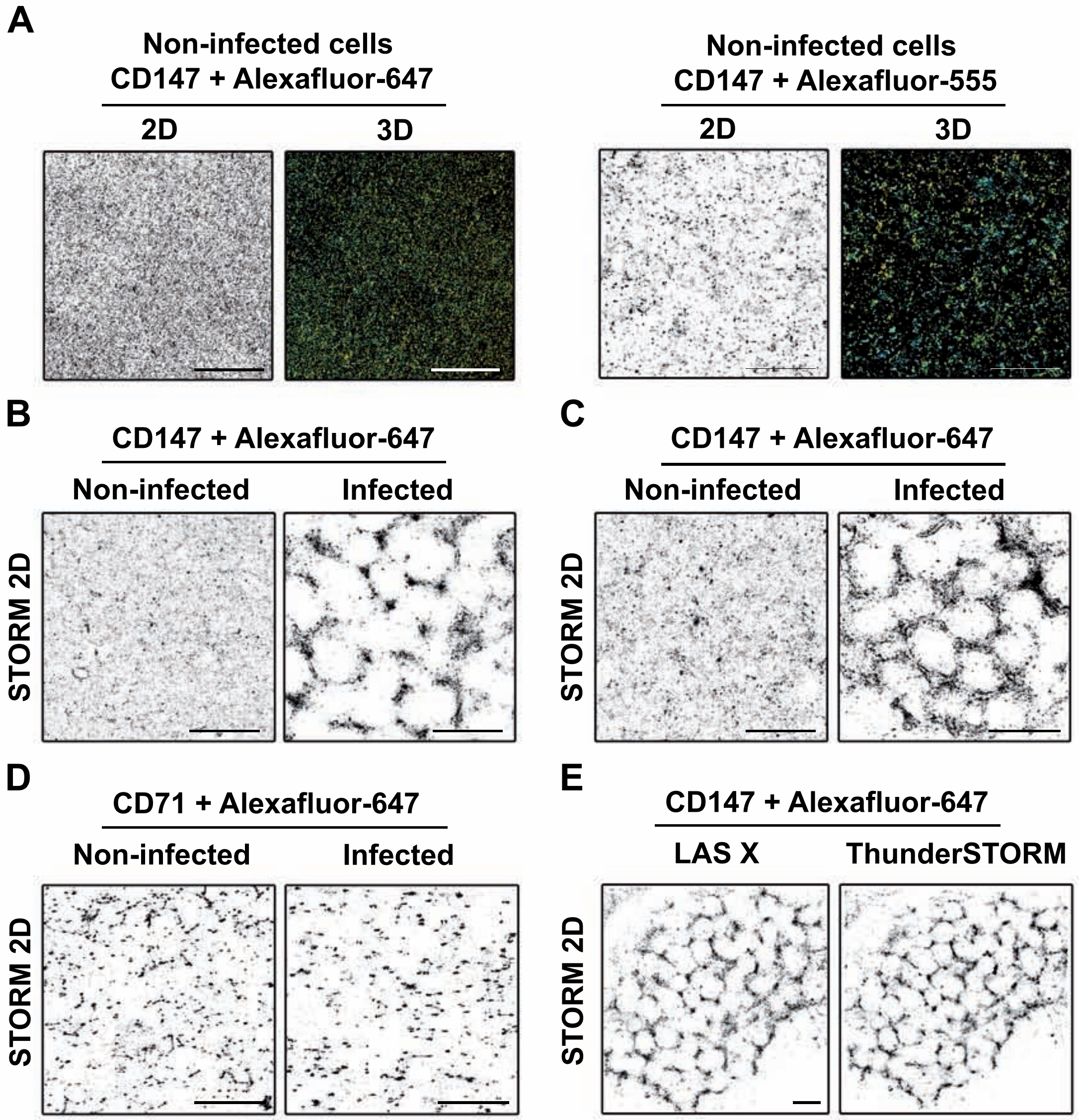

Supplementary Figure 9: 2D- and 3D-STORM control experiments.

(A) Shown are the 2D and 3D-STORM reconstructions of CD147 in non-infected HBMEC cells using two different fluorophores: Alexa-647 (left panels) and Alexa-555 (right panels), Vectashield mounting solution and the Leica LAS X 3D Visualization system (Scale bars 2.5 μm).

(B, C) Shown are the 2D reconstructions of CD147 in non-infected or infected HBMEC cells using two different STORM buffers: vectashield (B) or reducing buffer containing MEA (C) and the Leica LAS X 3D Visualization system (Scale bars 2.5 μm).

(D) Shown are the 2D reconstructions of a control receptor (transferrin Receptor, CD71) in non-infected or infected HBMEC cells. This receptor accumulates in clathrin-coated pits and is not recruited to bacterial adhesion sites. Images were acquired in vectashield and the Leica LAS X 3D Visualization system (Scale bars 2.5 μm).

(E) Shown are the 2D reconstructions of CD147 in non-infected or infected HBMEC cells acquired in vectashield and using the Leica LAS X 3D Visualization system (left) or ThunderSTORM (right) for image reconstructions.

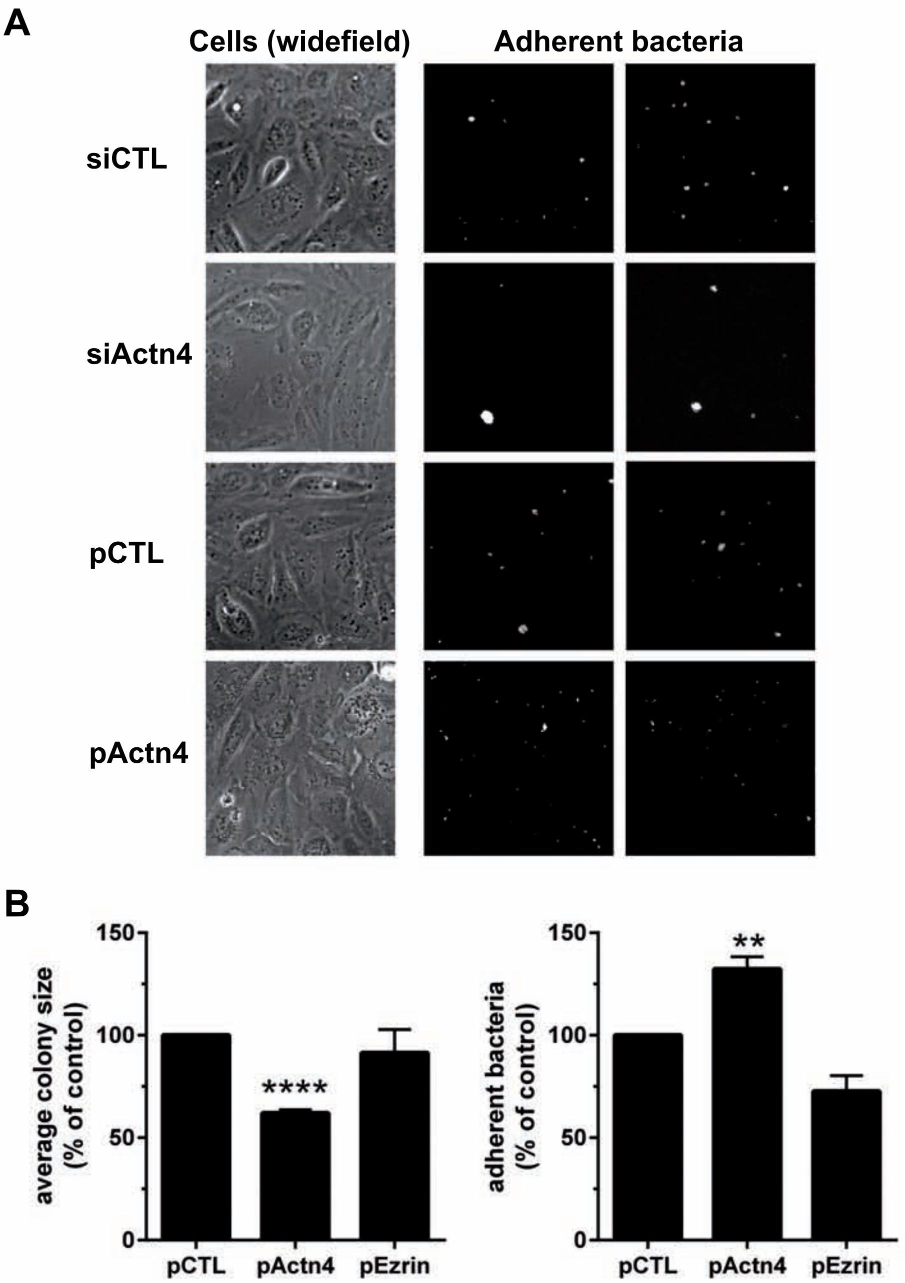

Supplementary Figure 10: Alpha-Actinin4, but not Ezrin, regulates meningococcal adhesion to human endothelial cells.

(A) Actn4 expression modulates bacterial adhesion to human endothelial cells under shear stress. HBMECs were transfected with control (siCTL) or Actn4 (siActn4) siRNA or with control (pCTL) or Actn4 (pActn4) plasmids. Shown are representative images of the adherent GFP-bacteria, following a 10 min infection under shear stress (0.04 dynes/cm2).

(B) Ezrin overexpression does not affect meningococcal adhesion under shear stress. HBMEC cells were transfected with plasmids encoding Actn4, Ezrin, or an empty plasmid as a control (pCTL). (Left) Number of adhesion events (meningococci adhering individually or in aggregates) on Actn4-depleted or Acnt4-overexpressing HBMEC cells following a 10 min infection under shear stress (0.04 dynes/cm2). **P <0.01, two-tailed Student's t-test. (Right) Average size (in pixels) of the meningococcal colony adhering to Actn4-depleted or Acnt4-overexpressing HBMEC cells. The number and the size of the adherent individual bacteria and/or bacterial aggregates were quantified using Image J software. (Mean ± s.e.m, n= 3, ****P< 0.0001).

Supplementary Figure 11:
Blots Figure 3A

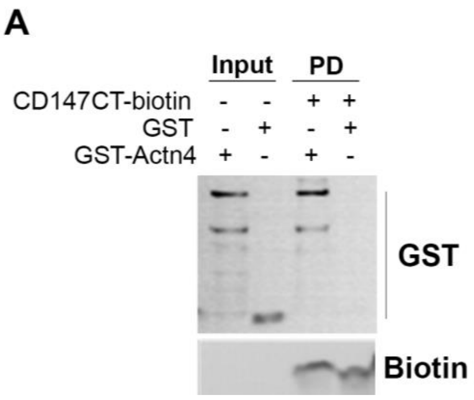

Gel ProSieve 14%

1-MW
2-PD GST
3-PD GST-actn4
4-input GST
5-input GST-actn4
6-output GST
7-output GST-actn4
8-input pept 1µg
9-PD GST
10-PD GST-actn4
11-input GST
12-input GST-actn4
13-output GST
14-output GST-actn4
15-MW

Lanes 2–7: 0,1µg GST or GST-actn4

Lanes 9–14: 0,2µg GST or GST-actn4

Blot 1 : anti GST (1/1000)1h

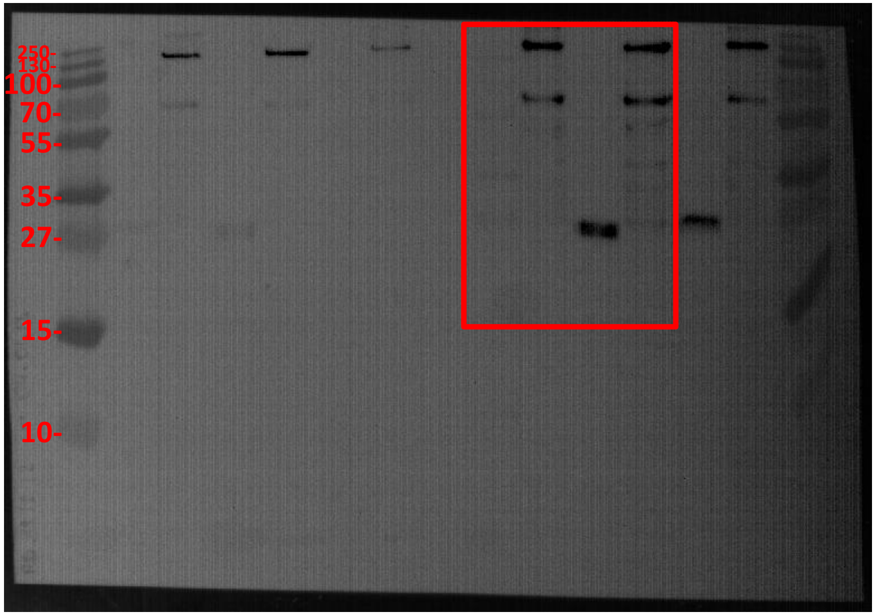

Blot 2 : anti biotin (1/1000) o/n (bas du gel)

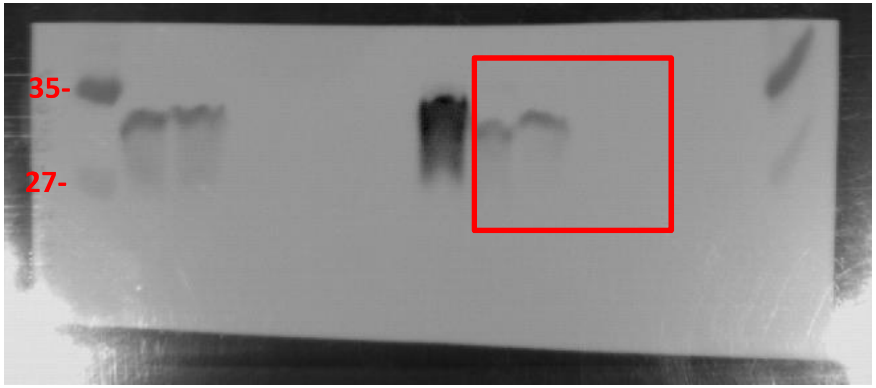

Supplementary Figure 12:
Blots figure 3C

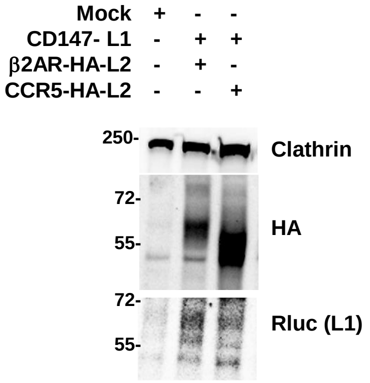

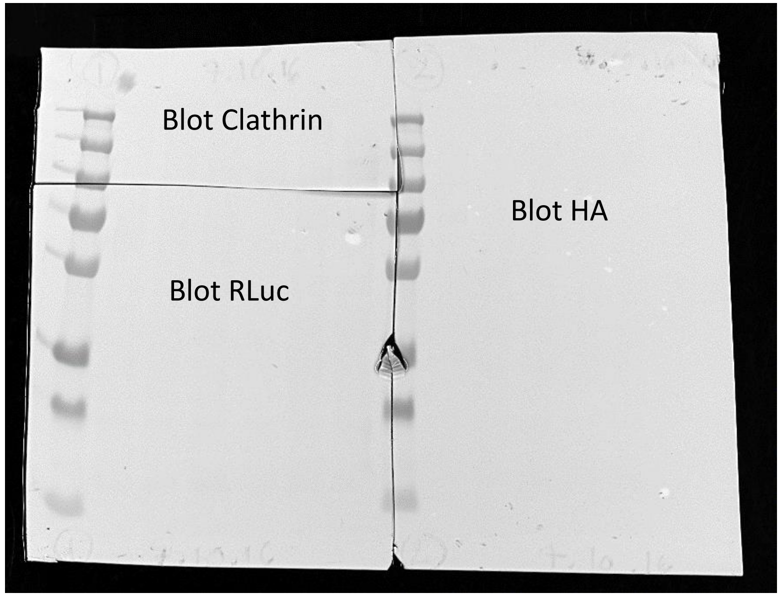

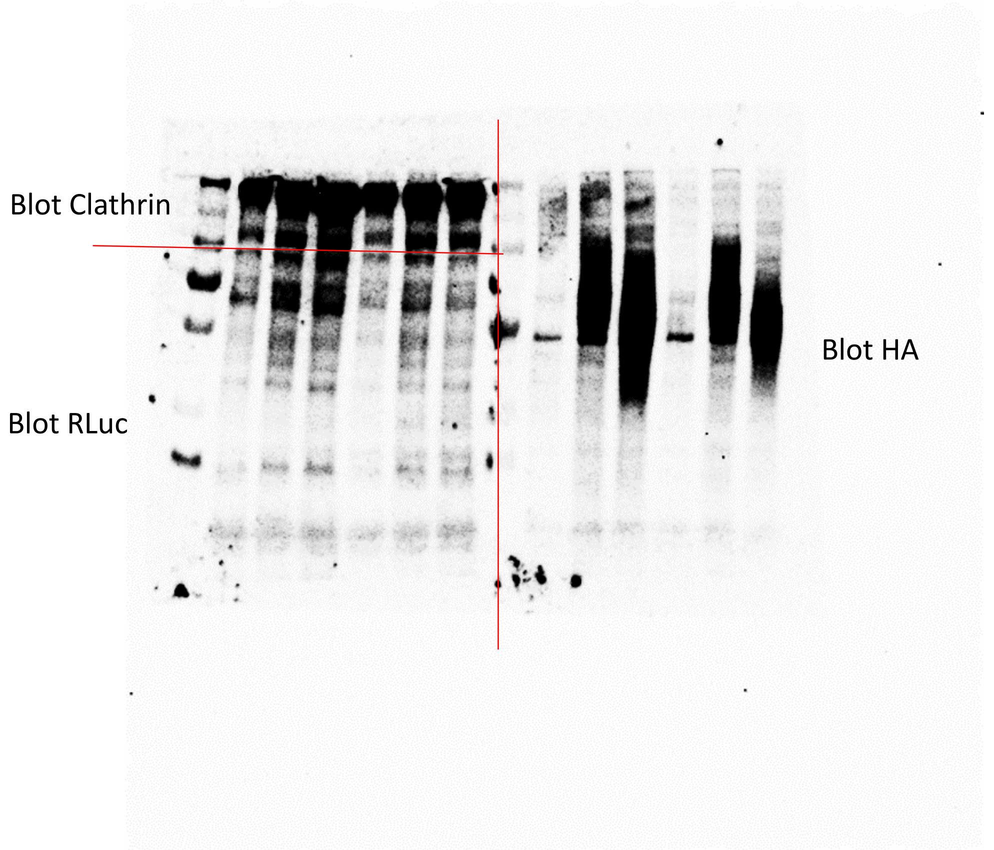

1- MW
2- MOCK
3- CD147-L1 + b2AR-HA-L2
4- CD147-L1 + CCR5-HA-L2
5- MOCK
6- CD147-L1 + b2AR-HA-L2
7- CD147-L1 + CCR5-HA-L2
8- MW
9- MOCK
10- CD147-L1 + b2AR-HA-L2
11- CD147-L1 + CCR5-HA-L2
12- MOCK
13- CD147-L1 + b2AR-HA-L2
14- CD147-L1 + CCR5-HA-L2

(Lanes 2–4: Exp 1; lanes 5–7: Exp 2; lanes 9–11: Exp 1; lanes 12–14: Exp 2)

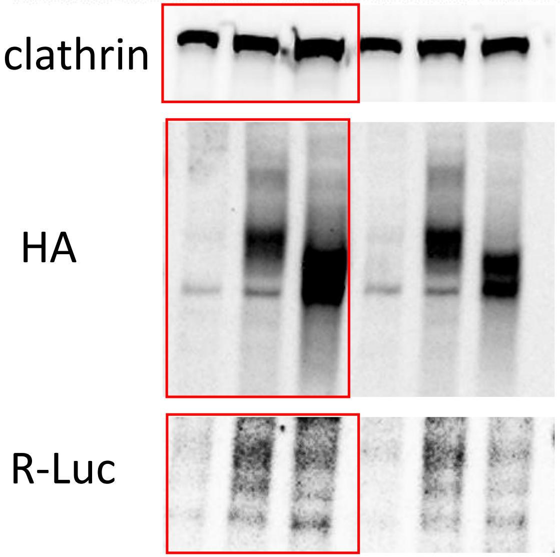

Supplementary Table 1: Antibodies used in this study

Target	*Reference*	*Provider*	*Condition of use*
Primary Antibodies			
CD147 (Basigin)	MEM-M6/1	AbD Serotec	IF= 1/100; IP= 2µg
HA	12CA5	Roche	IF= 1/100
Alpha-Actinin 4	ab108198	Abcam	IF=1/100 WB=1/1000
Clathrin	610500	BD transduction Lab	WB 1/1000
CD44	J173	Immunotech	IF=1/25
Ezrin	Serum	Dr Paul Mangeat	IF= 1/1000
GST	Serum	Dr Franck Perez	1/100
N. Meningitidis 2C4.3	Serum	Dr Xavier Nassif	IF= 1/1000
Pilin PilE	20D9	Dr Xavier Nassif	IF=1/1000 WB=1/10000
DAPI	D9542	Sigma Aldrich	300nM
Rhodamine-phalloidin	P1951	Sigma Aldrich	IF= 1/200
Secondary antibodies			
Goat Anti-Mouse CY3	115-165-146	Jackson ImmunoResearch	IF= 1/500
Goat Anti-Mouse CY5	115-175-146	Jackson ImmunoResearch	IF= 1/500
Goat Anti-Rabbit CY3	111-165-144	Jackson ImmunoResearch	IF= 1/500
Goat Anti-Rabbit CY5	111-175-144	Jackson ImmunoResearch	IF= 1/500
Goat Anti-Mouse Alexa Fluor®647	A-21235	Life technologies	IF= 1/500
Goat Anti-Mouse Alexa Fluor®555	A-21422	Life technologies	IF= 1/500

Supplementary Table 2: Primers used in this study

To introduce the HA tag into pcDNA3-CD147 vector:
CD147-HA Forward: 5' GTTCCAGATTACGCTTGAGGCAGGTGGCCCGAGGACG 3'
CD147-HA Reverse: 5' ATCGTATGGGTAGGAAGAGTTCCTCTGGCGG 3'

To delete the D1 domain of CD147:
CD147-del-D1 Forward: 5' CACGGGCCTCCCAGAGTGAAGGCTGTG 3'
CD147-del-D1 Reverse: 5' CCCGGAGGCTCCGTGGGTGCCCAGCAGC 3'

To introduce CD147 into pcDNA3.1 vector encoding the L1 fragment of Renilla Luciferase :
CD147-L1-HA Forward: 5' CCAGATTACGCTGCCGCCGGGCAACCCGGGAACGGC 3'
CD147-L1-HA Reverse: 5' AACATCGTATGGGTACATGGATCCATGGCGTCA 3'

To introduce B2AR into pcDNA3.1 vector encoding the L2 fragment of Renilla Luciferase :
B2AR-L2-HA Forward: 5'CCAGATTACGCTGCCGCCGGGCAACCCGGGAACGGC 3'
B2AR-L2-HA Reverse: 5' AACATCGTATGGGTACATGGATCCATGGCGTCA 3'

To introduce the YFP tag into pcDNA3-alpha-actinin-4 vector
ACTN4-YFP Forward : 5'AAATAAGCTTACCATGGTGGACTACCACGCGGCG 3'
ACTN4-YFP Reverse: 5' TTATTAAGCTTTGCCAGGTCGCTCTCGCCATA 3'
